# IPSE, a urogenital parasite-derived immunomodulatory protein, ameliorates ifosfamide-induced hemorrhagic cystitis through downregulation of pro-inflammatory pathways

**DOI:** 10.1038/s41598-018-38274-z

**Published:** 2019-02-07

**Authors:** Evaristus C. Mbanefo, Loc Le, Rebecca Zee, Nirad Banskota, Kenji Ishida, Luke F. Pennington, Justin I. Odegaard, Theodore S. Jardetzky, Abdulaziz Alouffi, Franco H. Falcone, Michael H. Hsieh

**Affiliations:** 1 Bladder Immunology Group, Biomedical Research Institute, Rockville, MD USA; 2 Division of Urology, Children’s National Medical Center, Washington, DC USA; 3 Department of Structural Biology, Stanford University School of Medicine, Stanford, CA USA; 4 Guardant Health, Redwood City, CA USA; 5 Life Science & Environment Sector, King Abdulaziz City for Science & Technology (KACST), Riyadh, Saudi Arabia; 6 Division of Molecular Therapeutics and Formulation, School of Pharmacy, University of Nottingham, Nottingham, UK; 7 Department of Urology, The George Washington University, Washington, DC USA

## Abstract

Ifosfamide and other oxazaphosphorines can result in hemorrhagic cystitis, a constellation of complications caused by acrolein metabolites. We previously showed that a single dose of IPSE (Interleukin-4-inducing principle from *Schistosoma* eggs), a schistosome-derived host modulatory protein, can ameliorate ifosfamide-related cystitis; however, the mechanisms underlying this urotoxicity and its prevention are not fully understood. To provide insights into IPSE’s protective mechanism, we undertook transcriptional profiling of bladders from ifosfamide-treated mice, with or without pretreatment with IPSE or IPSE-NLS (a mutant of IPSE lacking nuclear localization sequence). Ifosfamide treatment upregulated a range of proinflammatory genes. The IL-1*β*-TNF*α*-IL-6 proinflammatory cascade via NF*κ*B and STAT3 pathways was identified as the key driver of inflammation. The NRF2-mediated oxidative stress response pathway, which regulates heme homoeostasis and expression of antioxidant enzymes, was highly activated. Anti-inflammatory cascades, namely Wnt, Hedgehog and PPAR pathways, were downregulated. IPSE drove significant downregulation of major proinflammatory pathways including the IL-1*β*-TNF*α*-IL-6 pathways, interferon signaling, and reduction in oxidative stress. IPSE-NLS reduced inflammation but not oxidative stress. Taken together, we have identified signatures of acute-phase inflammation and oxidative stress in ifosfamide-injured bladder, which are reversed by pretreatment with IPSE. This work revealed several pathways that could be therapeutically targeted to prevent ifosfamide-induced hemorrhagic cystitis.

## Introduction

Hemorrhagic cystitis is a serious and difficult to manage complication resulting from exposure to certain chemotherapeutic agents^1^, radiation therapy^2^ and various viruses in immunosuppressed patients^3,4^. Indeed, anticancer doses of oxazaphosphorines, such as cyclophosphamide and ifosfamide, are limited in part due to the risks of this complication^1^. In the case of these agents, hepatic drug metabolism generates toxic acrolein which accumulates in bladder urine^5^. Fortunately, risks of chemotherapy-induced hemorrhagic cystitis have been decreased through the use of 2-mercaptoethane sulfonate Na (MESNA), which directly binds and neutralizes acrolein^6,7^. However, MESNA fails to treat established hemorrhagic cystitis^6,7^ and can also produce its own adverse reactions^8,9^. Other treatment options, including intravesically administered drugs^10,11^, systemically administered agents^6,12^, and nonpharmacological interventions^13,14^, are either investigational or feature significant potential side effects^5,15^.

The mechanisms underlying the initiation and pathogenesis of the acrolein-induced urotoxic effect are only partially elucidated. Knowledge gained from various studies and as reviewed by Haldar *et al*.^5^ has implicated pro-inflammatory, heme homeostasis, and oxidative stress response pathways in the pathogenesis of acrolein-triggered bladder damage. Accumulation of acrolein-containing urine in the bladder lumen depletes the mucosal glycosaminoglycan layer and the asymmetric unit membrane (uroplakin complex), exposing the urothelium. Acrolein induces pyroptosis in the urothelium, a highly inflammatory form of apoptosis. The resulting sloughing and denudation of the urothelial layer exposes the lamina propria, detrusor muscle, and the bladder vasculature to further damage^5^. Acrolein catalyzes reactions that generate reactive oxygen and nitrogen species (ROS and RNS) and superoxide radicals in the urothelium, resulting in membrane damage, DNA damage and cell death via the NF*κ*B pathway^16,17^. The activation and involvement of the inflammasomes complex in response to this oxidative stress results in the maturation and release of IL-1*β*, which in turn orchestrates a pro-inflammatory microenvironment in the urothelium^18^. This stress state also stimulates innate immune pattern recognition receptors (TLRs, NLRS and CLRs), sending signals that activate the NF*κ*B, STAT3, MAPK and other pro-inflammatory pathways, which lead to transcription of several pro-inflammatory cytokines (IL-1*β*, TNF*α* and IL-6), pro-inflammatory mediators (iNOS and COX-2) and chemokines that promote leukocyte infiltration and further drive inflammation and oxidative stress^18,19^. In response to hemorrhage from damaged blood vessels and accumulating superoxide radicals, the heme homeostasis pathway and the oxidative stress response pathways are fully activated via NRF2-mediated mechanisms^20^. The clotting, edema and constriction of the bladder results in hyperalgesia.

There is a significant need for additional approaches to prevent and treat chemotherapy-induced hemorrhagic cystitis. Some analogs of cyclophosphamide that may enhance cytostatic efficacy while limiting urotoxicity have been explored, albeit with limited success^21,22^. Candidate drugs targeting the inflammatory IL-1*β*, TNF*α* and IL-6 triad^18,23^ and/or promoting antioxidant responses show promise for ameliorating hemorrhagic cystitis but have not progressed beyond preclinical testing. Most efforts have been focused towards finding alternatives to MESNA, including anti-inflammatory molecules^24–26^, hemostatic agents^27,28^, antioxidants^29–31^, cytokines^12,32^, platelet rich plasma^33^, nutritional approaches^34^, and plant extracts^26,35^. These early-stage therapeutic candidates target pro-inflammatory pathways, heme homeostasis pathway and anti-oxidant homoeostasis.

Another potential approach to treat chemotherapy-induced hemorrhagic cystitis is to administer IL-4^12^, a potent anti-inflammatory cytokine known to antagonize the IL-1β, TNFα and IL-6 pathways. This finding led us to test and verify that a single dose of an IL-4-inducing, parasite-derived anti-inflammatory molecule (IPSE, the IL-4-inducing principle from *Schistosoma mansoni* eggs) ameliorated the inflammation, hemorrhage, and urothelial sloughing associated with ifosfamide-induced hemorrhagic cystitis^24^. IPSE binds immunoglobulins, notably IgE on the surface of basophils and mast cells, inducing secretion of preformed IL-4^36^. However, we suspect IPSE may have additional mechanisms underpinning its ability to alleviate ifosfamide-induced hemorrhagic cystitis. IPSE also sequesters chemokines^37^, which likely orchestrate anti-inflammatory responses. As an infiltrin possessing a nuclear localization sequence (NLS), IPSE is able to translocate into host cell nuclei to modulate host gene transcription^38^. Given that the transcriptional changes during ifosfamide-induced hemorrhagic cystitis are largely unknown, and because the underlying mechanisms of IPSE’s protective effects remain to be elucidated, we undertook transcriptome-wide profiling of the bladder of ifosfamide-treated mice using RNA-Seq. Furthermore, we studied the gene expression dynamics in IPSE pretreated mice challenged with ifosfamide. Using a mutant of IPSE lacking nuclear localization efficacy, we assessed the effect of the nuclear localization on IPSE therapeutic efficacy. Here, we show that key pro-inflammatory, heme homeostatic and oxidative stress response pathways are highly activated in the bladder following ifosfamide insult. Finally, we show that IPSE downregulates pro-inflammatory responses and contributes to restoring oxidative homeostasis as a potential protective mechanism, in addition to its involvement in promoting urothelial repair.

## Results

### Ifosfamide-induced hemorrhagic cystitis is ameliorated by IPSE

**Figure 1:**
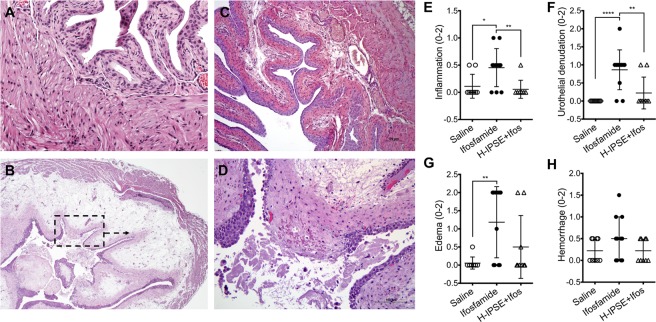
IPSE ameliorates ifosfamide-induced hemorrhagic cystitis. Mice were pretreated with saline or IPSE 24 hours before challenge with 400 mg/kg of ifosfamide. Bladders were assessed for histopathologic changes following ifosfamide insult in a blinded fashion. (**A**) Normal bladder showing intact urothelium with no signs of pathology. (**B**) Bladder from an ifosfamide-treated mouse (pretreated with saline) showing urothelial sloughing and edema. (**C**) Bladder from an IPSE-pretreated, ifosfamide-challenged mouse showing significant reduction in inflammation, urothelial denudation and edema. (**D**) High power view of bladder section shown in dotted box in (**B**). Graphs showing treatment group differences in bladder (**E**) inflammation, (**F**) urothelial denudation, (**G**) edema, and (**H**) hemorrhage. Each symbol represents the score for an individual mouse. Cross bar for each group denotes mean score. *p < 0.05, **p < 0.01, ****p < 0.001 based on post-hoc Students t-tests following significant difference among groups by ANOVA.

We recently showed that a single dose of IPSE was comparable to administration of recombinant IL-4 or three doses of MESNA in alleviating ifosfamide-induced hemorrhagic cystitis^24^. We used these established methods to obtain bladder samples for transcriptional profiling. Mice were administered: (1) saline or (2) IPSE, 24 hours before ifosfamide challenge, or (3) saline vehicle alone. Twelve hours following ifosfamide insult, bladder histopathology was analyzed in a blinded fashion. Compared to bladders from saline-treated mice (Fig. 1A), bladders from mice challenged with ifosfamide showed marked edema, dysregulated contraction, hemorrhage, and urothelial sloughing (Fig. 1B,D). Conversely, bladders from mice treated with IPSE before ifosfamide challenge were significantly protected from urothelial denudation and inflammation (Fig. 1C). Based on blinded scoring of bladder sections, we observed significant increases in inflammation (*p* = 0.0234), (Fig. 1E), urothelial denudation (*p* = 0.0001), (Fig. 1F), and edema (*p* = 0.0025), (Fig. 1G), and considerable increase in hemorrhage (Fig. 1H) in ifosfamide-treated mice. These features were markedly reduced in mice administered a single dose of IPSE before ifosfamide, in comparison to ifosfamide-treated mice. Both inflammation (*p* = 0.0092), and urothelial denudation (*p* = 0.0013), were significantly reduced (Fig. 1E,F), while edema and hemorrhage were considerable reduced (Fig. 1G,H). Taken together, these demonstrate characteristic features of ifosfamide-induced hemorrhagic cystitis, some of which were significantly reduced by IPSE pretreatment.

### Transcriptional profiles show massive pro-inflammatory response during ifosfamide-induced hemorrhagic cystitis

**Figure 2:**
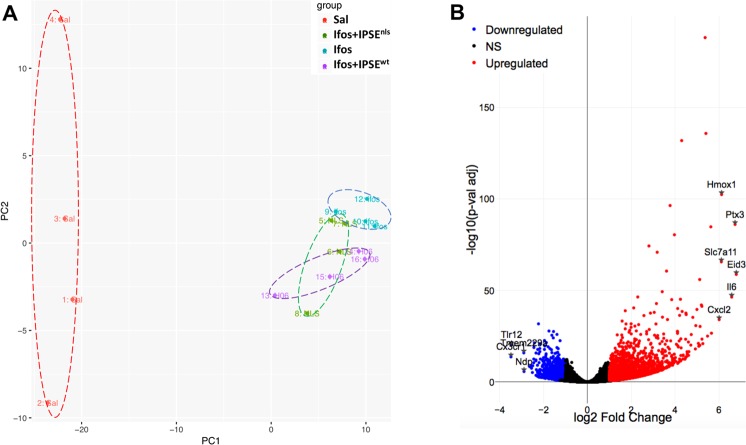
RNA-Seq analysis of ifosfamide-exposed bladders indicates multiple inflammation and stress response-related genes are differentially regulated. (**A**) Principal component analysis showed homogeneous clustering of gene expression among ifosfamide-treated mice (turquoise symbols labeled with “ifos”) and vehicle-treated mice (red symbols labeled with “Sal”). The PCA also showed no overlap of gene expression among ifosfamide-treated mice and IPSE-treated mice challenged with ifosfamide (purple symbols labeled with “H06”). However, there was overlap of gene expression among IPSE-treated mice and mice treated with the mutant of IPSE deficient for the nuclear localization sequence (green symbols labeled with “NLS”) (**B**) Volcano plots demonstrated upregulated and downregulated genes in bladders from ifosfamide-versus saline-treated mice. For this comparison, p-value (adjusted) <0.1 and log2(Fold Change) >1 (at least 2-fold) were applied as threshold values. NS: genes with non-significant changes in expression levels [black dots].

Ifosfamide is metabolized in the liver to generate acrolein, which accumulates in urine and damages the bladder^8^. To understand the transcriptional alterations elicited by acrolein in the bladder during ifosfamide-induced hemorrhagic cystitis, mice were treated with saline vehicle or ifosfamide. Gene expression dynamics in the ifosfamide-injured bladder were studied through RNA-Seq performed on bladders harvested 6 hours following ifosfamide injection. RNA sequencing was performed to a considerable depth (>20 million reads), more than 96% of which were successfully aligned to the *Mus musculus* genome (v. *Mm10*). Principal component analysis indicated gene expression homogeneity among ifosfamide-treated bladders relative to the vehicle control (Fig. 2A). Volcano plotting of differentially expressed genes and their associated statistical significance (Fig. 2B) revealed upregulation of a large set of genes (n = 2061) and downregulation of an appreciable number of genes (n = 1114), based on *p-value* (adjusted) <0.1 and *log2*(Fold Change) >1 (at least 2-fold). Among the top upregulated genes were *Il6*, a major member of the IL-1*β*, TNF*α* and IL-6 pro-inflammatory triad. Indeed, the *Il1b, Tnfa* and *Il6* genes were upregulated by about two orders of magnitude. These three cytokines together are major drivers of inflammatory responses. The *Ptx3* gene was also one of the top upregulated genes. The pentraxin protein family are major components of the humoral arm of innate immune response highly induced in response to inflammatory stimuli^39^. Chemokines were also highly upregulated, especially *Cxcl2* and *Ccl2*. The top activated upstream regulator is TNF*α* (Supplementary Fig. S1). In addition, the *Hmox1* gene encoding the heme oxygenase 1, the first enzyme of the heme oxygenase pathway, was also highly upregulated. The *Eid3* gene, also among the most upregulated genes, is involved in cellular responses to stress (Fig. 2B and Supplementary Fig. S1). The cysteine transporter, *Slc7a11*, which has been implicated in glutathione metabolism in the bladder^40^, was also significantly upregulated in ifosfamide-injured bladders (Fig. 2B).

**Figure 3:**
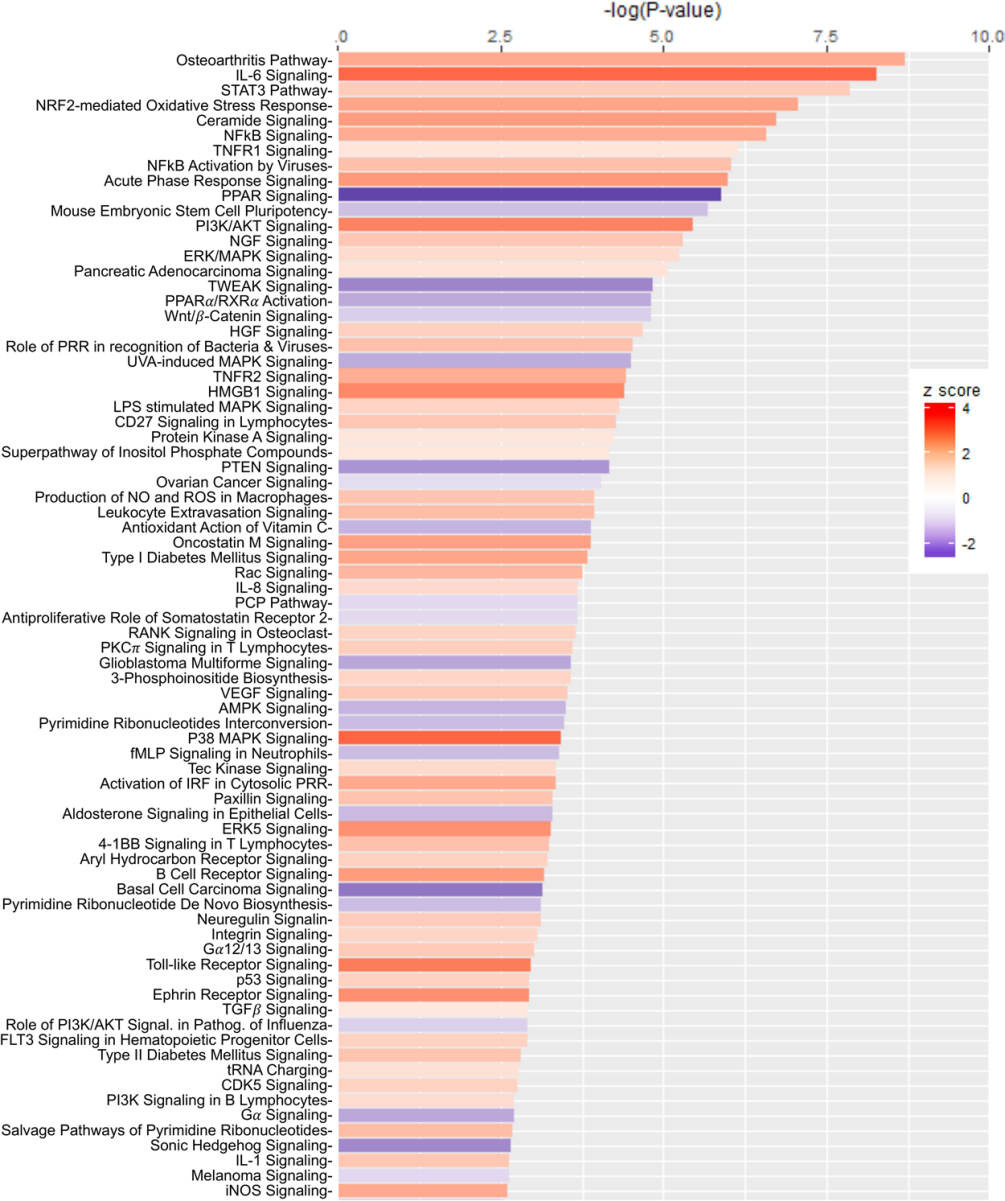
Most differentially altered pathways in the bladder during ifosfamide-induced hemorrhagic cystitis. Functional comparison of the transcriptome of bladders from ifosfamide- versus vehicle-treated mice was performed using IPA^57^. Bars are colored according to z-score (predicts activation or inhibition based on the degree of overlap between directional expressions from observed data and the QIAGEN-curated public knowledge base), with red showing activation and blue denoting inhibition. The size of each bar is proportional to its –log(p-value).

**Figure 4:**
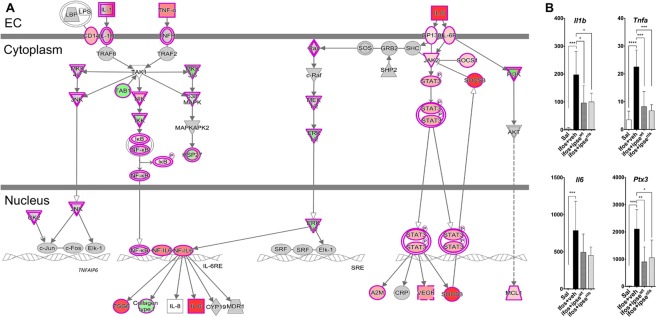
The IL-1*β*, TNF*α*, and IL-6 pathways, a major inflammatory pathway upregulated in the bladder during ifosfamide-induced hemorrhagic cystitis was downregulated by IPSE pretreatment. (**A**) Pathway and functional analyses were generated through the use of IPA^57^. Bladders of ifosfamide-exposed mice upregulated expression of genes from the IL-1*β*, TNF*α* and IL-6 pathways and their corresponding receptors and downstream nuclear transcriptional factors. In the case of IL-1*β* and TNF*α*, these cascades converge upon NF*κ*B. IL-6 also indirectly interacts with NF𝜅B through ERK1/2 activation of NF-IL6, which works with NF*κ*B to promote transcription of target genes. Keys: upregulation (red), downregulation (green), cytokines (square), growth factors (dotted square), phosphatase (triangle), kinases (inverted triangle), transmembrane receptors (ellipse), transcriptional regulators (wide circle), peptidase (rhombus), group or complex (double lined shapes), transporter (trapezium), acts on (line with filled arrow), translocate (line with open arrow), inhibition (line with perpendicular line at edge). (**B**) Il1b, Tnfa, Il6 and Ptx3 gene transcription were upregulated by 2–3 orders of magnitude in the bladders of ifosfamide-treated mice. Pretreatment with IPSE or its NLS mutant reduced the levels by ~50% relative to the ifosfamide-treated group. Similar trends were observed for cognate receptors and downstream transcription factors (data not shown).

Pathways and functional analyses expectedly revealed signatures of inflammation. Specifically, there was differential activity involving the IL-6 pathway, in which IL-1*β*, TNF*α* and IL-6 play major roles, and other pro-inflammatory pathways implicated in the pathogenesis of ifosfamide-induced hemorrhagic cystitis^18,23,41,42^ (Fig. 3 and Supplementary Fig. S1). The expression of *Il6* and its cognate receptors were highly upregulated, in addition to *Stat3* and the tyrosine protein kinase *Jak2*, which are both involved in IL-6 signaling (Fig. 4A and Supplementary Fig. S2A). Similarly, *Il1b*, *Tnfa* and their receptors were upregulated. These cascades converge through *Tak1* to promote formation of the I*κ*B-NF*κ*B complex and drive pro-inflammatory gene transcription in conjunction with NF-IL-6, the nuclear factor of IL-6 expression (Fig. 4A and Supplementary Fig. S2B). Accordingly, the STAT3 and NF*κ*B pathways, both major drivers of inflammation and immune response via the IL-1*β*-TNF*α*-IL-6 triad, were upregulated following ifosfamide insult transcription (Supplementary Fig. S2). Other major upregulated pro-inflammatory pathways and disease signaling cascades included those related to TNF receptors, iNOS, the acute phase response, osteoarthritis, diabetes mellitus, HMGB1, TREM1, oncostatin, generation of ROS and RNS, VEGF signaling, IL-8 signaling, leucocyte extravasation signaling and major innate immune-related cascades (Fig. 3 and Supplementary Fig. S3). Finally, there was noteworthy upregulation of the IL-17F-mediated allergic inflammatory signaling pathways (Supplementary Fig. S3).

### Transcriptional modulatory effects of IPSE on the ifosfamide-exposed bladder

**Figure 5:**
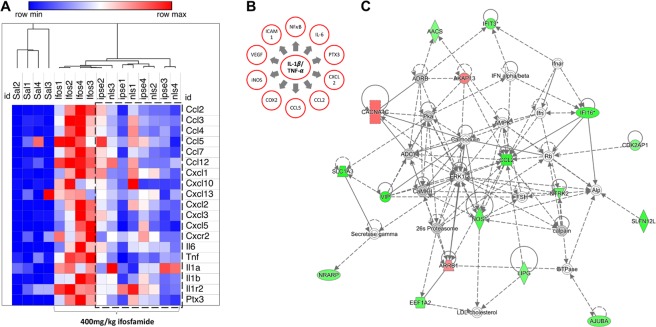
Effects of IPSE on bladder transcription of inflammation-related genes during ifosfamide-induced hemorrhagic cystitis. (**A**) Heat map showing levels of expression of genes encoding major proinflammatory proteins, their receptors, and downstream proinflammatory mediators and chemokines. Pretreatment with IPSE or its NLS mutant resulted in significant downregulation of genes encoding major proinflammatory proteins, their receptors, and downstream proinflammatory mediators and chemokines. Dotted line show downregulation in mice pretreated with IPSE or its NLS mutant. Red designates degrees of gene upregulation while blue denotes downregulation. The columns represent data for individual mice in each treatment group with “one minus Pearson correlation” method of hierarchical clustering (“Sal”: saline vehicle treatment, “Ifos”: ifosfamide treatment only, “ipse”: ifosfamide and IPSE treatment, “nls”: ifosfamide and IPSE NLS mutant treatment). (**B**) Schematic representation of the relationships among the IL-1*β*, TNF*α* and IL-6 triad and downstream pro-inflammatory cytokines and mediators. (**C**) A representative predicted mechanistic network, generated using IPA^57^, of gene interactions showing inhibitory relationships among chemokines (Ccl2), several interferon-induced proteins and nitric oxide synthase. Green color signifies directional gene expression that is part of dataset, passed the cut off values and downregulated. Orange color signifies directional gene expression that is part of dataset, passed the cut off values and upregulated. Gray color signifies gene expression that is part of dataset but unchanged. White color signifies genes that is not part of dataset. For other keys to the shape annotations, see description in Fig. 4.

We recently reported that IPSE, an immunomodulatory protein of parasite origin, can ameliorate much of the pathology associated with ifosfamide-induced hemorrhagic cystitis^24^(and Fig. 1). To provide insight into the underlying mechanisms of the IPSE’s protective effects, we undertook gene expression profiling of the ifosfamide-challenged bladder, with or without IPSE pretreatment. Mice were treated with saline, IPSE or the NLS mutant of IPSE (lacking nuclear localization but retaining other functions), 24 hours before challenge with ifosfamide. Gene expression dynamics were profiled through RNA-Seq analysis of bladders harvested 6 hours following ifosfamide administration. Compared to mice receiving ifosfamide without IPSE pretreatment, genes encoding cytokines driving pro-inflammatory responses (*Il1b, Tnfa* and *Il6* triad) were downregulated 50% in the bladders of mice treated with IPSE or its NLS mutant before ifosfamide challenge (Figs 4B and 5A). While *Il1b* (*p* = 0.0213) and *Tnfa* (*p* = 0.0007) were significantly downregulated, *Il6* was considerably downregulated as well (Fig. 4B). Similar downward trends in gene expression were observed for genes encoding these cytokines’ receptors, chemokines and downstream transcriptional factors driving inflammation (Fig. 5A). The relationship between *Il1b, Tnfa* and *Il6* triad and the downstream regulators and mediators is illustrated in Fig. 5B. Interestingly, the expression of *Cxcl10* (IP-10), a major interferon gamma-induced chemokine, was highly increased in ifosfamide-injured bladders but downregulated in the bladders of IPSE or its NLS mutant-pretreated, ifosfamide-exposed mice (Fig. 5A). The transcriptional levels of several other chemokines also involved in inflammatory responses and recruitment of cells to sites of stress were highly upregulated in bladders only exposed to ifosfamide, but conversely downregulated in ifosfamide-injured bladders pretreated with IPSE or IPSE mutant lacking the nuclear localization function (Fig. 5A). The predicted mechanistic network of gene interaction grids associated with IPSE administration featured a network linking downregulation of *Ccl2* (and other chemokines genes) to the downregulation of genes, including genes encoding a number of gamma interferon-inducible proteins and nitric oxide synthase (Fig. 5C and Supplementary Fig. S4). Other mechanistic networks suggesting IPSE induced downregulation of additional pro-inflammatory factors were also noted (Supplementary Fig. S4).

**Figure 6:**
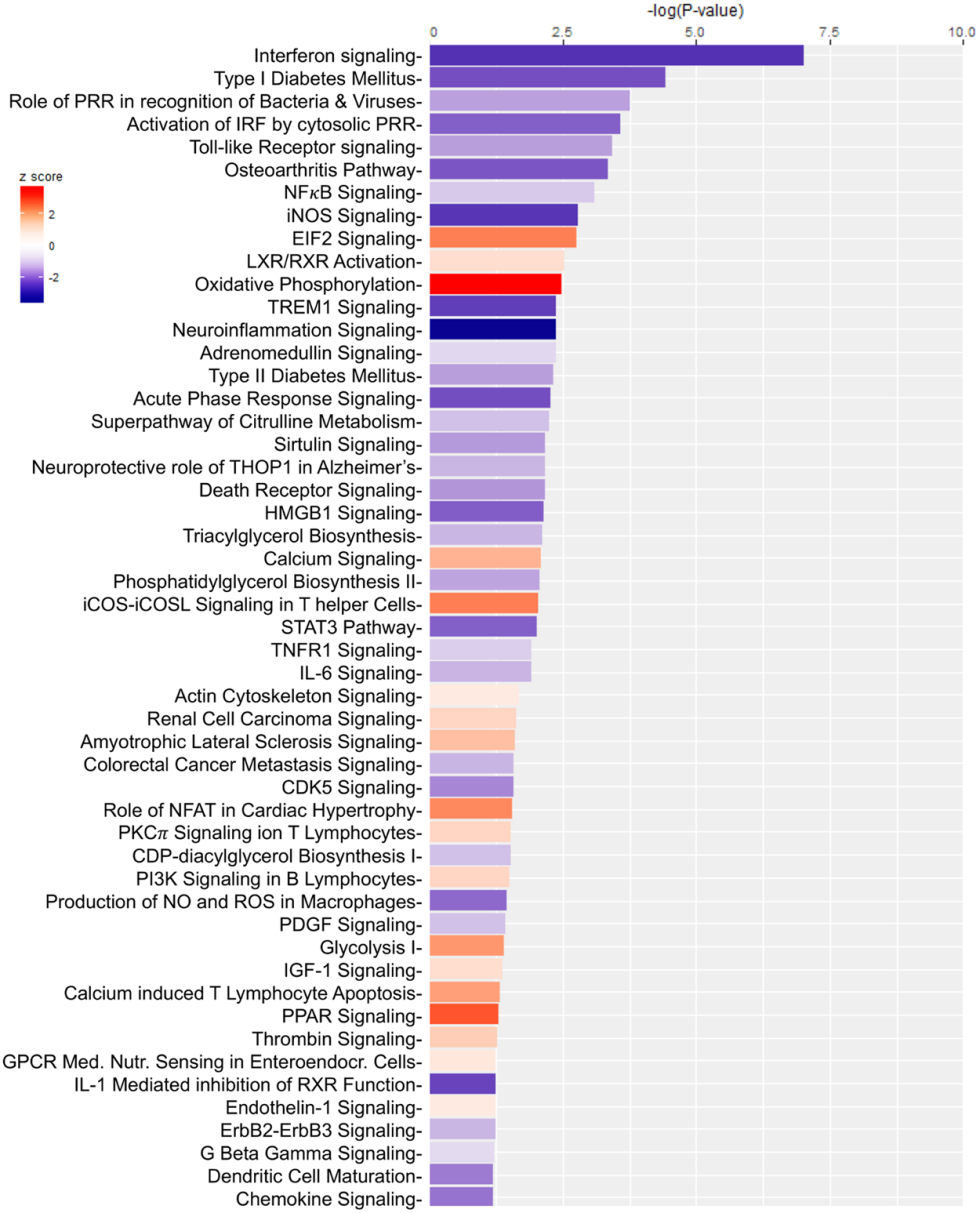
Most differentially altered gene expression pathways in bladders from IPSE-pretreated mice challenged with ifosfamide. Mice were pretreated with saline or IPSE, 24 hours before challenge with 400 mg/kg of ifosfamide. The bladders were subjected to transcriptional profiling (RNA-Seq), followed by pathway and functional analyses generated using IPA^57^. Bars are colored according to z-score (predicts activation or inhibition based on the degree of overlap between directional expressions from observed data and the QIAGEN-curated public knowledge base), with red showing activation and blue denoting inhibition. The size of each bar is proportional to its –log(p-value).

The most downregulated gene pathway (in terms of statistical significance) in the bladders of IPSE-pretreated, ifosfamide-exposed mice, compared to bladders exposed only to ifosfamide, was the interferon signaling pathway (Fig. 6). Many of the major gene pathways noted in Fig. 3 to be highly upregulated in ifosfamide-damaged bladders were relatively downregulated in bladders pretreated with IPSE before ifosfamide challenge (Fig. 6). Similar profile of downregulation of inflammatory pathways was observed in the bladders of mice pretreated with the NLS mutant of IPSE before ifosfamide challenge (Supplementary Fig. S5A). These downregulated pathways included those related to interferon signaling, inflammatory diseases such as osteoarthritis and diabetes mellitus, pattern recognition receptor signaling pathways of the innate immune system, pro-inflammatory pathways including NF*κ*B, iNOS, neuroinflammation, TREM1, the acute phase response, HMGB1, STAT3, IL-6, TNFR, IL-1 and pathways involved in the production of ROS and RNS (Fig. 6). We also observed a relative increase in metabolic gene expression relevant to oxidative phosphorylation, glycolysis and PPAR signaling. PPAR signaling has also been shown to be anti-inflammatory^43–45^. Interestingly, the most downregulated genes in terms of z-score were those related to the neuro-inflammation signaling pathway (astrocytes and microglia). While a direct effect of IPSE on bladder neurons could not be inferred based on this finding, due to a lack of astrocytes and microglia in the bladder, we have observed significant reduction in bladder pain in IPSE-treated mice challenged with ifosfamide^24^. Head to head comparison of gene expression dynamics from mice pretreated with IPSE and its NLS mutant before ifosfamide challenge showed differential upregulation of extracellular matrix related pathways, including integrin signaling, ILK (integrin-linked kinase) signaling, actin cytoskeleton signaling, paxillin signaling and downregulation of cholesterol biosynthesis pathway (Supplementary Fig. S5B). Although these pathways are unrelated to inflammation and oxidative stress, the comparison indicated relative upregulation of some inflammation related pathways albeit to a less extent, including leucocyte extravasation signaling, VEGF signaling, IL-8 and chemokine signaling, and some chemokine receptor signaling (Supplementary Fig. S5B).

### Oxidative stress responses during ifosfamide-induced hemorrhagic cystitis and the effect of IPSE

**Figure 7:**
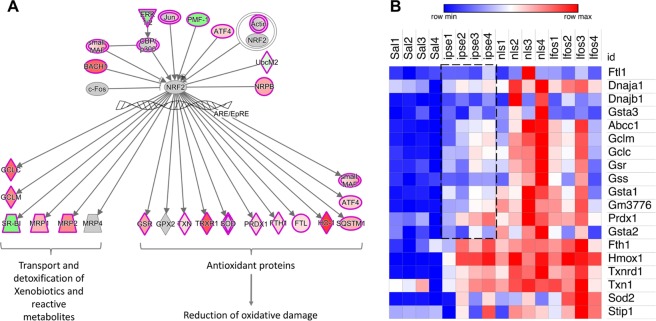
Oxidative stress responses of the bladder during ifosfamide-induced hemorrhagic cystitis. Pathway and functional analyses were generated through the use of IPA^57^. (**A**) Schematic representation of the relationships between NRF2 and antioxidant proteins and proteins involved in heme homeostasis and xenobiotic detoxification. A more detailed version is shown in Supplementary Fig. S6. The keys to the shapes and colors are as detailed in Fig. 4. (**B**) Heat map showing levels of expression of genes encoding major antioxidant enzymes. Compared to the ifosfamide only group and group pretreated with the NLS mutant of IPSE, there were relative reduction in the expression of Abcc1, Gclc, Gclm, Gsr, Gss, Gsta1, Gsta2, Gm3776 and to some extent Prdx1, Stip1, Sod2, Dnaja1 and Dnajb1. Red designates gene upregulation while blue denotes downregulation. The columns represent data for individual mice in each treatment group without hierarchical clustering (“Sal”: saline vehicle treatment, “ipse”: ifosfamide and IPSE treatment, “nls”: ifosfamide and NLS mutant of IPSE treatment, “Ifos”: ifosfamide treatment only).

Another hallmark of ifosfamide-induced hemorrhagic cystitis is a significant oxidative stress response to acrolein exposure and resulting hemorrhage^17^. Our data underscores a major role for the erythroid-derived leucine zipper NRF2 as a nuclear factor involved in regulating oxidative stress responses to ifosfamide injury of the bladder^28,29,46^ (Figs 3 and 7 and Supplementary Fig. S6). The NRF2-mediated oxidative stress responses pathway, which regulates the expression of antioxidant and heme homeostatic enzymes, was one of the most upregulated pathways in the bladder following ifosfamide insult (Fig. 3). We noted considerable upregulation of genes encoding enzymes including heme oxygenase (*Hmox1*), which catalyzes the first step in heme homeostasis, the antioxidant thioredoxin reductase (*Txnrd1*), which catalyzes the reduction of thioredoxin to restore redox homeostasis, peroxiredoxin (*Prdx1*), which detoxifies peroxide radicals, and glutathione reductase (*Gsr*), which reduces glutathione disulfide to glutathione, an important antioxidant that scavenges hydroxyl radicals. Also upregulated were thioredoxin (*Txn1*), superoxide dismutase (*Sod*) and ferritin heavy chain protein (*Fth1*), which are involved in redox signaling, superoxide partitioning and iron homeostasis, respectively (Fig. 7B). Genes encoding proteins involved in xenobiotic detoxification were also upregulated in response to acrolein (Fig. 7B). In addition, the p38 MAPK pathway, implicated in responses to stress stimuli^25^, was also substantially upregulated. The antioxidant action of Vitamin C pathway was also downregulated.

Compared to bladders exposed only to ifosfamide or pretreated with the NLS mutant of IPSE before ifosfamide, bladders pretreated with IPSE before ifosfamide relatively downregulated gene expression of some antioxidant enzymes, including *Abcc1, Gclc, Gclm, Gm3776*, *Gsr, Gss, Gsta1, Gsta2, Prdx1, Sod2, Stip1*, *Dnaja1* and *Dnajb1* (Fig. 7B). It is notable that the downregulated gene expression of proteins in this pathway were mainly those involved in DNA damage sensing, glutathione-mediated redox homeostasis, superoxide partitioning and detoxification of xenobiotics, and metal ion homeostasis (Fig. 5B). In particular, relatively lower expression of genes encoding superoxide dismutase (*Sod2*) and the ferritin protein (*Fth1)* suggest relatively earlier restoration of iron homeostasis in IPSE pretreated mice, supporting the observed decrease in bladder hemorrhage induced by IPSE pretreatment before IFS challenge^24^. Downregulation of the stress induced protein (*Stip1*) suggest reduced oxidative stress. Downregulation of proteins involved in DNA damage sensing suggest decreased pyroptosis and tissue damage. Taken together, these data suggest in IPSE-pretreated bladders exposed to ifosfamide, that there is a significant anti-oxidant response following accumulation of acrolein, but lower expression of genes related to detoxification of xenobiotics, DNA damage sensing and iron homeostasis suggest earlier restoration of oxidative homeostasis compared to the group treated with the NLS mutant of IPSE or ifosfamide only.

### Other pathways altered during ifosfamide-induced hemorrhagic cystitis

The most downregulated pathway in the ifosfamide-exposed bladder was the peroxisome proliferator-activated receptor (PPAR) signaling pathway, which is involved in lipid homoeostasis^47^, in addition to its anti-inflammatory effect^43,44^ and role in the development and maintenance of IL-4-dependent alternatively activated status in macrophages^45^ (Fig. 3 and Supplementary Fig. S7). Interestingly, PPAR signaling was relatively increased in IPSE pretreated mice compared to mice administered ifosfamide only (Fig. 6). TWEAK signaling, Wnt/*β*-catenin and Hedgehog signaling, which can mediate anti-inflammatory responses, were likewise downregulated in the ifosfamide only group (Fig. 3). The aldosterone signaling pathway in epithelial cells, which is involved in ion transport to maintain electrolyte and water balance across epithelial surfaces, was also downregulated (Fig. 3). Finally, analysis of diseases and functions affected by bladder ifosfamide challenge showed considerable upregulation of functions related to organismal injury and abnormalities, inflammatory diseases, cancer, cell proliferation, cellular movement and hematological development and function (Supplementary Fig. S8). There was also notable upregulation of genes and regulators in the neuroinflammatory pathways (Supplementary Fig. S9). Analysis of differentially altered genes in the neuroinflammatory pathways indicated significant expression of genes encoding proinflammatory cytokines and mediators in the astrocytes, microglia, including their effect on post-synaptic neuron (Supplementary Fig. S9). Conversely, there was downregulation of the neuroinflammatory cascades in the IPSE pretreated group (Fig. 6), in addition to our previous observation of IPSE-induced reduction in ifosfamide-related bladder pain^24^. Although this may suggest an ameliorative role for IPSE in the acrolein-associated hyperalgesia, the presence of astrocytes or microglia in the peripheral nervous system within the bladder is unlikely. Finally, HIF-1*α*-mediated hypoxia-related signaling cascade was upregulated in the bladder after ifosfamide insult, consistent with the hemorrhage associated with hemorrhagic cystitis (Supplementary Fig. S10).

## Discussion

Herein we describe the first transcriptome-wide profiling of the bladder during ifosfamide-induced hemorrhagic cystitis. To accomplish this, we used a tractable mouse model which recapitulates the pathogenesis of hemorrhagic cystitis^12^ resulting from the urotoxic effect of acrolein, a byproduct of ifosfamide metabolism. This study has verified a number of important findings regarding specific biological aspects of ifosfamide-induced hemorrhagic cystitis. We have expanded upon this body of work by defining multiple key pathogenetic mechanisms through comprehensive transcriptomics. Furthermore, our RNA-Seq data extends our prior work on the therapeutic effect of IPSE in ifosfamide-induced hemorrhagic cystitis^24^. This study has revealed a central role played by the IL-1*β*, TNF*α* and IL-6 triad in driving the substantial inflammation associated with ifosfamide-induced hemorrhagic cystitis^18,23,41,42^. A 100-fold increase in expression of *Il1b*, *Tnfa* and *Il6* was reduced ~50% by pretreatment with a single dose of IPSE or a mutant of IPSE lacking the nuclear localization function (IPSE NLS mutant). We also confirmed that gene members of the heme hemostasis and oxidative stress response biological systems were highly transcribed following ifosfamide-induced hemorrhagic cystitis, presumably to restore antioxidants to normal levels^27–31^. The level of upregulation of these genes expressing antioxidant molecules were relatively lower in the IPSE group as compared to ifosfamide only group or IPSE NLS mutant group, indicating limiting effect of IPSE on oxidative stress. Thus, we have shown that these urotoxicity-associated transcriptional changes, especially inflammatory responses and to a less extent oxidative stress, were downregulated by IPSE when administered before ifosfamide challenge.

Unlike MESNA, which binds to and neutralizes acrolein directly to prevent urotoxicity, IPSE prevents or reverses the inflammatory changes that drive bladder damage following ifosfamide exposure. We postulate that IPSE, through its inhibitory transcriptional effects on IL-1*β*, TNF*α* and IL-6, key upstream cytokines driving inflammation via NF*κ*B and STAT3, limits ifosfamide-triggered inflammation, urothelial denudation and vascular pathogenesis. The ability of IPSE to induce gene expression of uroplakins^24^, crucial urothelial barrier function genes, is a likely contributor to IPSE’s therapeutic effect on the bladder following chemical insult^48^. Other candidate drugs for ifosfamide-induced hemorrhagic cystitis have also been shown to specifically target the IL-1*β*-TNF*α*-IL-6 pathway. Recombinant IL-4, quinovic acid glycosides, anakinra, diallyl disulfide and other anti-inflammatory candidates were separately shown to reduce the pathogenesis of hemorrhagic cystitis by inhibition of the expression of these inflammatory cytokines and their receptors^12,23,25,26^. In accord with our observations, other drug studies have identified a therapeutic requirement for downregulation of IL-1*β*, TNF*α* and IL-6 associated transcriptional factors (NF*κ*B and STAT3)^16,25^ and downstream inflammatory mediators (iNOS and COX-2)^12,16,41,42,49^.

Besides effects on the IL-1*β*, TNF*α* and IL-6 triad, IPSE may also mediate critical gene expression changes in chemokines during ifosfamide-induced hemorrhagic cystitis. A range of major chemokines genes (*Ccl2*, *Ccl3, Ccl4, Ccl5, Ccl7, Ccl12, Cxcl1, Cxcl2, Cxcl3, Cxcl5, Cxcl10 and Cxcl13)* were significantly downregulated by pretreatment with either IPSE or its NLS mutant before ifosfamide insult. These chemokines act as chemo-attractants for immune cells to sites of inflammation during stress, tissue injury or infection. Notably, *Cxcl10* transcription was upregulated in the bladder following ifosfamide insult, but was significantly decreased by comparison in IPSE-pretreated, ifosfamide-exposed bladders. Indeed, CXCL10 (interferon gamma-induced protein 10 - IP-10) blockade has been previously shown to significantly dampen cyclophosphamide-induced hemorrhagic cystitis^50^. Interleukin 8 receptor (*Cxcr2)* was also relatively downregulated by IPSE pretreatment before ifosfamide challenge. Notably, *Cxcr2* was previously identified to play an important role in cyclophosphamide induced hemorrhagic cystitis^51^.

IPSE likely orchestrates a portion of its therapeutic effects through actions on other cytokines. Gene expression network analysis revealed a link between the observed downregulation of CCL2 in the bladders of IPSE-pretreated, ifosfamide-challenged mice (versus the bladders from mice receiving only ifosfamide) and several gamma interferon-inducible proteins. This coincides with the upregulation of CXCL10 (interferon gamma-induced protein 10 - IP-10) as well. Also, the interferon signaling pathway was the most downregulated pathway in the bladders of IPSE-pretreated mice as compared to bladders only exposed to ifosfamide. Our results point to an association between downregulation of the interferon signaling pathway and amelioration of acrolein-induced urotoxicity, a mechanism that has not been previously linked to protection from hemorrhagic cystitis. The interferon signaling pathway has been previously shown to cross-talk with inflammasomes activated during inflammatory responses to irritants^52,53^. Some of these gamma interferon inducible genes are in turn linked to the development of pyroptosis, a highly inflammatory form of programmed cell death (Fig. 6B). Acrolein-induced pyroptotic cell death is a major determining factor of the severity of ifosfamide-induced urotoxicity^5,16^. Pyroptosis can be compounded by activation of the inflammasomes complex, which generates reactive species which perpetuate a vicious cycle of cell death^19^. The ability of IPSE to downregulate the interferon pathway, in conjunction with downregulation of major inflammatory pathways, may limit inflammasomes activation and thus reduce acrolein-induced pyroptotic cell death. We hypothesize that IPSE, by bringing these processes to heel, subsequently modulates downstream urothelial damage, hemorrhage, oxidative stress and cellular infiltration.

The therapeutic efficacy of IPSE in ifosfamide-induced hemorrhagic cystitis may also partially depend on its modulation of oxidative stress cascades. Acrolein is a potent inducer of oxidative stress^17^. Indeed, the NRF2-mediated oxidative stress responses pathway, which restores heme homeostasis and antioxidant responses, was highly expressed in the setting of ifosfamide-induced hemorrhagic cystitis^46^. Accordingly, *Hmox1* and *Slc7a11* were among the top upregulated genes from this transcriptomic analysis. *Slc7a11* is a cysteine transporter, which has been implicated in glutathione metabolism in the bladder^40^. NRF2 induces the expression of heme oxygenase 1 (*Hmox1*), the first enzyme of the heme oxygenase pathway, and several antioxidant enzymes including glutathione reductase (*Gsr*), thioredoxin (*Txn*), thioredoxin reductase (*Txnrd1*), superoxide dismutase (*Sod*), peroxiredoxin 1 (*Prdx1*), ferritin light chain (*Ftl1*) and ferritin heavy chains (*Fth*1)^20^. An association between an increase in the expression of NRF2 and protection from ifosfamide-induced hemorrhagic cystitis is consistent with a previous report linking hemostasis to reduction in hemorrhagic cystitis^28,29,46^. In addition, there is a strong pathophysiological relationship between inflammation and oxidative stress^54^. Severe pyroptosis can lead to enzymatic tissue damage and cellular DNA damage, which generate reactive species and superoxide radicals that induce oxidative stress^5,54^. When the bladder vasculature is exposed and injured following inflammation-driven urothelial damage, the resulting hemorrhage and release of heme further promotes oxidative stress. Based on the rationale that limiting inflammation reduces oxidative stress, and restoration of oxidative homeostasis initiates tissue repair processes, hemostatic agents and antioxidants have been widely tested as alternative therapies for preventing or reducing ifosfamide-induced hemorrhagic cystitis^27–31,46^. The link between inflammation and oxidative stress is evident from findings that most antioxidants showing efficacy in ifosfamide-induced hemorrhagic cystitis also downregulate pro-inflammatory cytokines and their downstream mediators^29,31^. In the same vein, anti-inflammatory drug candidates with efficacy in ifosfamide-induced hemorrhagic cystitis can also restore antioxidant enzyme activity to homoeostatic levels^25^.

In this study in relation to the foregoing, we observe relative transcriptional downregulation in oxidative stress responses by IPSE pretreatment but not for pretreatment with the NLS mutant of IPSE. It was interesting to observe that the gene encoding the proteins involved in iron homeostasis (*Fth1*) was relatively restored to baseline in bladders from the IPSE treated group. Also, genes encoding some antioxidant proteins involved in xenobiotic detoxification and hemostasis (*Gclm, Gclc, Gsr, Gss, Gsta1, Gsta2* and *Gm3776*), superoxide detoxification (*Sod2*) and stress induced chaperones (*Stip1, Dnaja1* and *Dnajb1*) were also reduced in bladders from the IPSE-pretreated group but not in the ifosfamide only or IPSE NLS mutant group. In addition, gene encoding the multidrug resistance protein 1 (*Abcc1, Mrp1*), which functions as an anion transporter with glutathione as a substrate^55^, returned to basal levels in the ifosfamide-exposed bladder following IPSE pretreatment. Taken together, these differences suggest reduced levels of oxidative stress are present in ifosfamide-exposed bladders from IPSE-pretreated group, evident in reduced levels of genes related to detoxification of xenobiotics, DNA damage sensing, hemostasis and iron homeostasis. Although the IPSE NLS mutant lacking the nuclear localization function partially downregulated inflammation, it did not show similar level of downregulation of oxidative stress. The ability to translocate into the nucleus arms IPSE with important transcriptional modulatory property which may be limited for the NLS mutant. However, this mutant of IPSE potentially still retains its other extracellular and extra-nuclear functions.

Ifosfamide injury of the bladder may disrupt homeostasis of pathways besides oxidative stress. The PPAR pathway was downregulated in the ifosfamide-exposed bladder, which would presumably impair restoration of lipid homeostasis following epithelial membrane damage by acrolein. The PPAR pathway is also a modulator of inflammatory responses^43,44^ in addition to its role in the development and maintenance of IL-4 dependent alternatively activated status in macrophages^45^, which we speculate may be inhibited in the highly inflammatory environment of the ifosfamide-injured bladder. Interestingly, relative to the ifosfamide only group, we observe upregulatory effect of IPSE pretreatment on transcription of gene members of the PPAR pathway. The most IPSE-downregulated genes in the ifosfamide-exposed bladder were those related to the neuro-inflammation signaling pathway (specifically, pathways active in central nervous system cells, i.e., astrocytes and microglia). We have previously reported that IPSE alleviates ifosfamide-induced bladder pain by assessment of allodynia^24^. It remains to be shown whether this observed downregulation of neuro-inflammatory signaling is directly linked to this protective effect on allodynia, especially given the absence of astrocytes and microglia in the bladder.

While this study has revealed potential mechanistic changes associated with ifosfamide-induced hemorrhagic cystitis, and presented evidence of possible underlying mechanisms of IPSE’s therapeutic effect, our RNA-Seq-based approach cannot establish a causal relationship between observed gene expression and phenomena of interest (including IPSE’s therapeutic effects). Given that pretreatment with the NLS mutant of IPSE partially downregulated inflammation as wildtype IPSE but not oxidative stress different from wild type IPSE, this dataset did not completely elucidate whether the therapeutic effects of IPSE are due to its IL-4-inducing properties, chemokine sequestration, or nuclear translocation-related direct transcriptional effects. However, there is indication that the NLS function is at least required for the effect of IPSE on oxidative homoeostasis. The downregulation of interferon signaling and its related genes is intriguing, but it is unclear to which extent this can be ascribed to IPSE-induced IL-4. Also, RNA-Seq does not capture epigenetic or post-translational regulation of gene or protein expression and activity. Moreover, we focused on a single, early time point following ifosfamide exposure. Although we observed differential transcription of multiple genes of interest at this time point, it is unlikely that this cross-sectional analysis has captured all relevant gene expression changes. For instance, IPSE in the context of being the most abundant schistosomes secreted protein is the major driver of Th2 response, however, this early time point study design has focused on only non-T-cell, non-B-cell sources of IPSE-induced IL-4 release^36,56^. Further studies may be required to show whether similar therapeutic effect will be observed when the adaptive response sources of IL-4 are involved. Finally, this study focused on transcriptional changes in the bladder alone and did not examine systemic gene expression induced by ifosfamide and IPSE. It is possible that such gene expression may account for some of ifosfamide and IPSE’s *in vivo* effects.

In conclusion, we have elucidated transcriptional dynamics associated with ifosfamide-induced hemorrhagic cystitis. These data provide new insights into the underlying mechanisms driving acrolein-induced urotoxicity associated with the use of ifosfamide and other oxazaphosphorines. We also showed that IPSE, an anti-inflammatory, parasite-derived molecule with therapeutic potential for ifosfamide-induced hemorrhagic cystitis^24^, downregulates major inflammatory pathways and to some extent oxidative stress, potentially related to its mechanisms of effect. Our work demonstrates that there may be therapeutic potential for naturally occurring anti-inflammatory molecules, including pathogen-derived factors, as alternative or complementary therapies for ifosfamide-induced hemorrhagic cystitis. Apart from inhibition of inflammation and modest restoration of normal levels of antioxidants by IPSE but not its NLS mutant, we did not observe complete prevention of acrolein-induced oxidative stress by IPSE pretreatment. This is probably due to IPSE’s inability to directly bind to and neutralize acrolein (the mechanism of MESNA). Thus, IPSE is playing only a limited role on oxidative stress while suppressing inflammation. However, we have only compared one dose of IPSE given before ifosfamide challenge against three doses of MESNA^24^. It remains to be shown whether IPSE will produce more ameliorative effects when given in multiple doses or through alternative routes. Ongoing work is focusing on optimization of IPSE, specifically related to its IL-4 induction and chemokine binding properties, to enhance its efficacy while preventing toxicity. Our hope is that these variations on IPSE and its administration will result in significantly improved efficacy, and ultimately, an alternative to MESNA in preventing ifosfamide-induced hemorrhagic cystitis.

## Materials and Methods

### Ethical approval

Animal experiments reported in this study were conducted in a humane manner, adhering to relevant U.S. and international guidelines. Our animal handling and experimental protocols were reviewed and approved by the Institutional Animal Care and Use Committee (IACUC) of the Biomedical Research Institute, Rockville, Maryland, USA. Our IACUC guidelines comply with the U.S. Public Health Service Policy on Human Care and Use of Laboratory Animals.

### Animals, reagents and drugs

Female 7-week-old C57BL/6 mice (Charles River Laboratories, Wilmington, MA, USA) were housed using 12-h light-dark cycles in temperature-controlled holding rooms, with an unlimited supply of dry mouse chow and water. Ifosfamide (>98% purity) was purchased from Sigma-Aldrich (Sigma-Aldrich, St. Louis, MO, USA). IPSE cloning, expression and purification was performed as previously described^24,38^.

### Ifosfamide-induced hemorrhagic cystitis model

The ifosfamide-induced hemorrhagic cystitis model presented in this study was performed following methods previously described by Macedo *et al*.^12^. Mice were intravenously injected with 25 μg of either IPSE or a mutant of IPSE lacking the nuclear localization sequence (IPSE NLS mutant) or saline 24 hours before intraperitoneal ifosfamide injection (400 mg/kg). Mice were then monitored for 6 hours post-ifosfamide injection before they were sacrificed for downstream experiments. Bladders were aseptically collected for RNA purification.

### RNA purification

RNA was isolated from mouse bladders using TRIzol Reagent and PureLink RNA Mini Kit (Invitrogen), according to manufacturers’ instructions. Briefly, aseptically excised bladders were homogenized in 1 ml TRIzol Reagent by bead-beating using ceramic beads (Omni International) and a mini-bead beater (Biospec). Following a 5-min incubation, 0.2 ml chloroform was added and again incubated for 3 min before centrifugation at 12,000 × g for 15 min to separate homogenates into aqueous and organic phases. The aqueous supernatant (~400ul) was mixed with an equal volume of 70% ethanol before binding the mixture to RNA binding columns by centrifugation. On-column DNase digestion (Invitrogen) was performed for 30 minutes, following the manufacturer’s protocols. After column washes and drying, RNA was eluted in RNase-free water, quantified and its quality checked using a NanoDrop 1000 spectrophotometer (Thermo Scientific) and Bioanalyzer 2100 (Agilent).

### RNA sequencing and RNA-seq analysis pipeline

RNA sequencing was performed using the Illumina-HiSeq. 4000 NGS platform at a depth of >20 million reads. Analyses were conducted using the RNA analysis tools of the Galaxy platform (www.usegalaxy.org). Raw sequence reads were aligned to the mouse genome (*Mm10*) by HISAT2. The resulting alignment files, along with the corresponding mouse genome annotation file, were used as the input for HTSeq-count. DESeq. 2 was used to determine differentially expressed genes between each pair of treatment groups. PCA plots were also generated by DESeq. 2. The DESeq. 2 results files containing gene IDs, log2 fold change and standard deviation, p-values and adjusted p-values were processed further downstream for functional analysis.

### Functional and pathway analysis, statistics and plots

Pathway, mechanistic network and functional analyses were generated through the use of IPA (QIAGEN Inc., https://www.qiagenbio- informatics.com/products/ingenuity-pathway-analysis)^57^. The threshold cut-off was set at *p-value* (adjusted) <0.1 for gene expression comparisons between bladders exposed to ifosfamide versus saline vehicle, and *p-value* < 0.05 for gene expression comparisons between IPSE-pretreated, ifosfamide-exposed bladders versus bladders only exposed to ifosfamide. The cut off for log2(fold change) was set at >1 (2 fold). The heatmaps were generated using the Morpheus program from Broad Institute. Other data analyses and plots were generated using GraphPad Prism v 6.00, and *ggplot2* and *plotly* packages in R. For comparisons among groups, one way analysis of variance (ANOVA) was performed and if significant, was followed by *post hoc* Student *t*-tests for pairwise comparisons after confirming a normal distribution. Plotted data show individual data points with error bars representing means and standard deviation.

### Histology

Bladders were fixed in 10% neutral-buffered formalin and later dehydrated and embedded in paraffin. Paraffin-embedded bladders were cut into 5 micron sections and then processed for hematoxylin and eosin staining. The stained sections were evaluated microscopically (in a blinded fashion by J.I.O.) for the presence of urothelial denudation, lamina propria edema, hemorrhage, and cellular infiltration.

## Supplementary information

Supplementary Figures

## Data Availability

All raw and analyzed data are readily available on request.
