# Supplementary material for: IPSE, a urogenital parasite-derived immunomodulatory protein, ameliorates ifosfamide-induced hemorrhagic cystitis through downregulation of pro-inflammatory pathways
Source: Sci Rep. 2019 Feb 7;9:1586. doi: 10.1038/s41598-018-38274-z (PMC6367514; doi:10.1038/s41598-018-38274-z)
Supplement: Supplementary file 1 — Supplementary Figures [file 41598_2018_38274_MOESM1_ESM.pdf]

IPSE, a urogenital parasite-derived immunomodulatory protein, ameliorates ifosfamide-induced hemorrhagic cystitis through downregulation of pro-inflammatory pathways

Evaristus C. Mbanefo[1,2], Loc Le[1], Rebecca Zee[1,2], Nirad Banskota[1], Kenji Ishida[1], Luke F. Pennington[3], Justin I. Odegaard[4], Theodore S. Jardetzky[3], Abdulaziz Alouffi[5], Franco H. Falcone[6], Michael H. Hsieh[1,2,7*]

Affiliations

[1]Bladder Immunology Group, Biomedical Research Institute, Rockville, MD, USA
[2]Division of Urology, Children's National Medical Center, Washington, DC, USA
[3]Department of Structural Biology, Stanford University School of Medicine, Stanford, CA, USA
[4]Guardant Health, Redwood City, CA, USA
[5]Life Science & Environment Sector, King Abdulaziz City for Science & Technology (KACST), Saudi Arabia
[6]Division of Molecular Therapeutics and Formulation, School of Pharmacy, University of Nottingham, UK.
[7]Department of Urology, The George Washington University, Washington, D.C., USA

INGENUITY
PATHWAY ANALYSIS

Analysis Name: Ifos_vs_Sal p0.05- 2018-05-10 06:13 PM
Analysis Creation Date: 2018-06-01
Build version: 470319M
Content version: 43605602 (Release Date: 2018-03-28)

Supplementary Fig. S1. Summary of the Pathways, Functional and Network analysis using Ingenuity Pathway Analysis (see ref. 57). This file shows top 5 each of canonical pathways, upstream regulators, diseases and disorders, molecular and cellular functions, physiological system development and functions, tox functions (hepatotoxicity, nephrotoxicity and cardiotoxicity), regulator effect networks, mechanistic networks, top 10 upregulated and downregulated genes.

Analysis Settings

Reference set: Ingenuity Knowledge Base (Genes Only)

Relationship to include: Direct and Indirect

Includes Endogenous Chemicals

Optional Analyses: My Pathways My List

Filter Summary:

Consider only relationships where

confidence = Experimentally Observed

(c) 2000-2018 QIAGEN. All rights reserved.

Top Canonical Pathways

Name	p-value	Overlap
Molecular Mechanisms of Cancer	6.54E-11	45.4 % 179/394
Role of Macrophages, Fibroblasts and Endothelial Cells in Rheumatoid Arthritis	1.87E-10	47.0 % 147/313
Osteoarthritis Pathway	1.98E-09	49.5 % 105/212
IL-6 Signaling	5.41E-09	54.7 % 70/128
Death Receptor Signaling	5.60E-09	59.1 % 55/93

Top Upstream Regulators

Upstream Regulator	p-value of overlap	Predicted Activation
TNF	5.90E-29	Activated
beta-estradiol	4.64E-24	Activated
TGFB1	8.46E-23	Activated
PDGF BB	2.89E-21	Activated
NFKBIA	1.14E-19	Activated

Top Diseases and Bio Functions

Diseases and Disorders

Name	p-value	#Molecules
Cancer	9.96E-09 - 3.77E-157	6084
Organismal Injury and Abnormalities	9.96E-09 - 3.77E-157	6207
Gastrointestinal Disease	5.18E-09 - 2.21E-134	5630
Hepatic System Disease	4.36E-09 - 5.09E-44	3964
Reproductive System Disease	9.38E-09 - 2.35E-31	3788

Molecular and Cellular Functions

(c) 2000-2018 QIAGEN. All rights reserved.

INGENUITY PATHWAY ANALYSIS

Name	p-value	#Molecules
Cell Death and Survival	7.49E-09 - 5.07E-46	2138
Cellular Movement	8.05E-09 - 2.03E-34	1415
Gene Expression	4.12E-12 - 2.97E-26	1469
Cell Cycle	1.06E-08 - 5.19E-24	937
Cellular Development	1.04E-08 - 1.75E-23	1982

Physiological System Development and Function

Name	p-value	#Molecules
Organismal Survival	7.31E-11 - 3.36E-58	1638
Organismal Development	5.68E-09 - 1.52E-42	2357
Tissue Morphology	9.09E-09 - 4.09E-37	1581
Hematological System Development and Function	9.09E-09 - 2.20E-28	1267
Cardiovascular System Development and Function	7.49E-09 - 2.20E-25	921

Top Tox Functions

Assays: Clinical Chemistry and Hematology

Name	p-value	#Molecules
Increased Levels of Alkaline Phosphatase	3.01E-01 - 5.09E-07	46
Increased Levels of ALT	2.57E-01 - 1.97E-03	20
Increased Levels of AST	3.01E-01 - 2.13E-03	14
Increased Levels of Creatinine	2.21E-01 - 2.82E-03	26
Increased Levels of Red Blood Cells	1.32E-02 - 1.32E-02	42

Cardiotoxicity

(c) 2000-2018 QIAGEN. All rights reserved.

Name	p-value	#Molecules
Cardiac Infarction	5.11E-01 - 4.35E-06	117
Cardiac Necrosis/Cell Death	6.58E-01 - 6.01E-06	120
Cardiac Arrythmia	1.00E00 - 7.57E-06	119
Cardiac Enlargement	6.58E-01 - 9.71E-06	183
Cardiac Arteriopathy	5.81E-01 - 1.62E-04	149

Hepatotoxicity

Name	p-value	#Molecules
Liver Hyperplasia/Hyperproliferation	1.00E00 - 6.99E-38	3855
Liver Necrosis/Cell Death	5.11E-01 - 3.88E-11	147
Liver Proliferation	5.11E-01 - 4.65E-10	114
Hepatocellular Carcinoma	6.58E-01 - 7.21E-08	439
Liver Cirrhosis	5.11E-01 - 1.00E-04	115

Nephrotoxicity

Name	p-value	#Molecules
Renal Proliferation	6.58E-01 - 8.00E-10	136
Renal Necrosis/Cell Death	6.58E-01 - 2.88E-08	239
Renal Damage	6.58E-01 - 1.50E-06	124
Glomerular Injury	1.00E00 - 1.41E-04	131
Renal Hypoplasia	5.11E-01 - 2.13E-04	36

Top Regulator Effect Networks

ID	Regulators	Diseases & Functions	Consistency Score
1	C3	Interaction of leukocytes	4.364
2	trinitrobenzenesulfonic acid	Chemotaxis of myeloid cells	4.041

(c) 2000-2018 QIAGEN. All rights reserved.

INGENUITY PATHWAY ANALYSIS

3	TAC1	Recruitment of blood cells	3.9
4	MIF	Migration of granulocytes	3.881
5	C5AR1	Homing of cells	3.873

Top Networks

ID	Associated Network Functions	Score
1	Vitamin and Mineral Metabolism, Cell Cycle, Cellular Assembly and Organization	23
2	Hereditary Disorder, Ophthalmic Disease, Organismal Injury and Abnormalities	23
3	Connective Tissue Disorders, Developmental Disorder, Hereditary Disorder	23
4	Gene Expression, Auditory and Vestibular System Development and Function, Cancer	23
5	Cell-To-Cell Signaling and Interaction, Cellular Movement, Hematological System Development and Function	23

Top Tox Lists

Name	p-value	Overlap
Renal Necrosis/Cell Death	1.25E-11	43.5 % 239/550
Liver Necrosis/Cell Death	5.88E-11	47.6 % 147/309
Liver Proliferation	3.63E-10	49.2 % 118/240
Increases Liver Hyperplasia/Hyperproliferation	7.65E-09	54.8 % 68/124
Increases Renal Proliferation	1.16E-07	50.7 % 75/148

Top Analysis-Ready Molecules

Expr Log Ratio up-regulated

Molecules	Expr. Value	Expr. Chart
**EID3**	↑ 6.792	
**PTX3**	↑ 6.737	
**IL6**	↑ 6.583	
**HMOX1**	↑ 6.122	
**SLC7A11**	↑ 6.111	

(c) 2000-2018 QIAGEN. All rights reserved.

INGENUITY PATHWAY ANALYSIS

CXCL3	↑ 6.011
TMEM236	↑ 5.660
ADAMTS4	↑ 5.632
LSMEM1	↑ 5.482
Mt2	↑ 5.407

Expr Log Ratio down-regulated

Molecules	Expr. Value	Expr. Chart
CX3CR1	↓ -3.487	
Tlr12	↓ -3.468	
TMEM229A	↓ -2.901	
NDP	↓ -2.891	
CYP1A1	↓ -2.633	
FZD10	↓ -2.549	
2010016I18Rik	↓ -2.544	
C130021I20Rik	↓ -2.508	
WSCD1	↓ -2.494	
CLEC12A	↓ -2.450	

(c) 2000-2018 QIAGEN. All rights reserved.

INGENUITY PATHWAY ANALYSIS

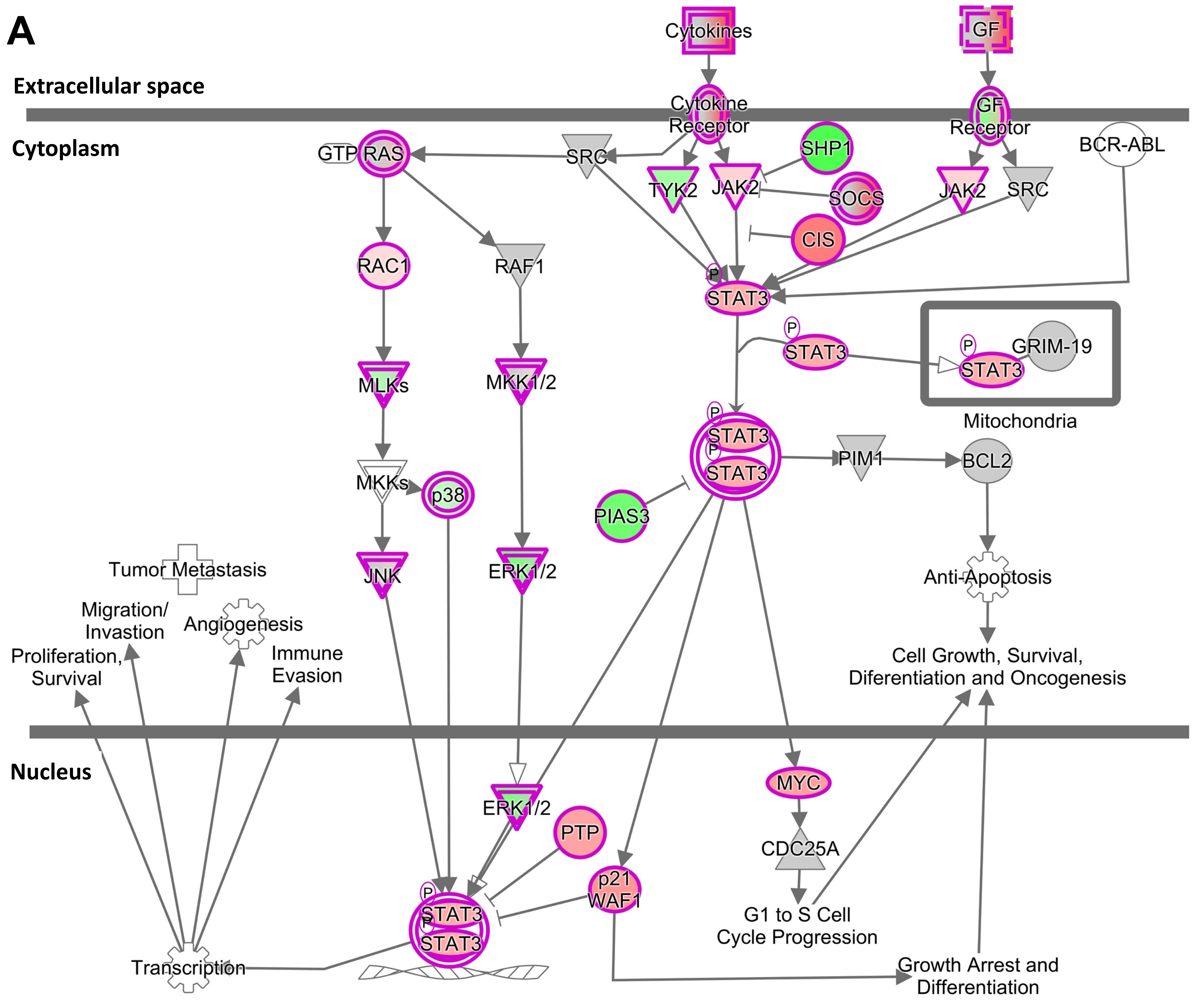

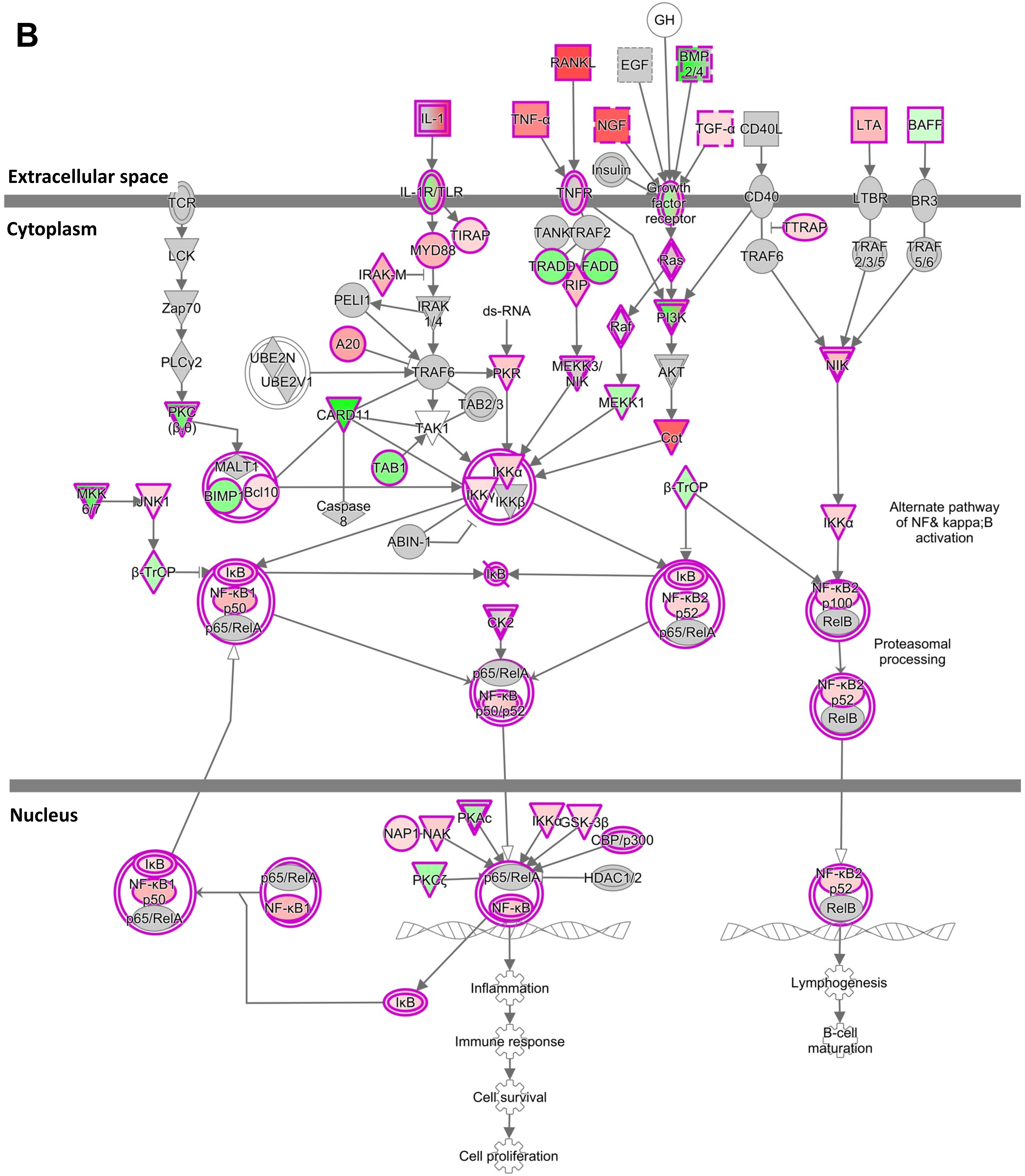

Supplementary Fig. S2. Major upregulated pro-inflammatory pathways during ifosfamide induced hemorrhagic cystitis. Pathways, Functional and Network analyses were generated using IPA[57]. Following ifosfamide injection and acrolein induced urotoxicity, there was upregulation of upstream cytokines (IL-6, IL-1β and TNFα), their receptors, adaptor proteins, protein kinases and nuclear transcriptional factor (STAT3 and NFκB) in the **(A)** STAT3 pathway and **(B)** NFκB pathway. Keys: upregulation (red), downregulation (green), cytokines (square), growth factors (dotted square), phosphatase (triangle), kinases (inverted triangle), transmembrane receptors (ellipse), transcriptional regulators (wide circle), peptidase (rhombus), group or complex (double lined shapes), transporter (trapezium), acts on (line with filled arrow), translocate (line with open arrow), inhibition (line with perpendicular line at edge).

Supplementary Fig. S3. Pg 1. Role of IL-17F in Allergic Airway Diseases

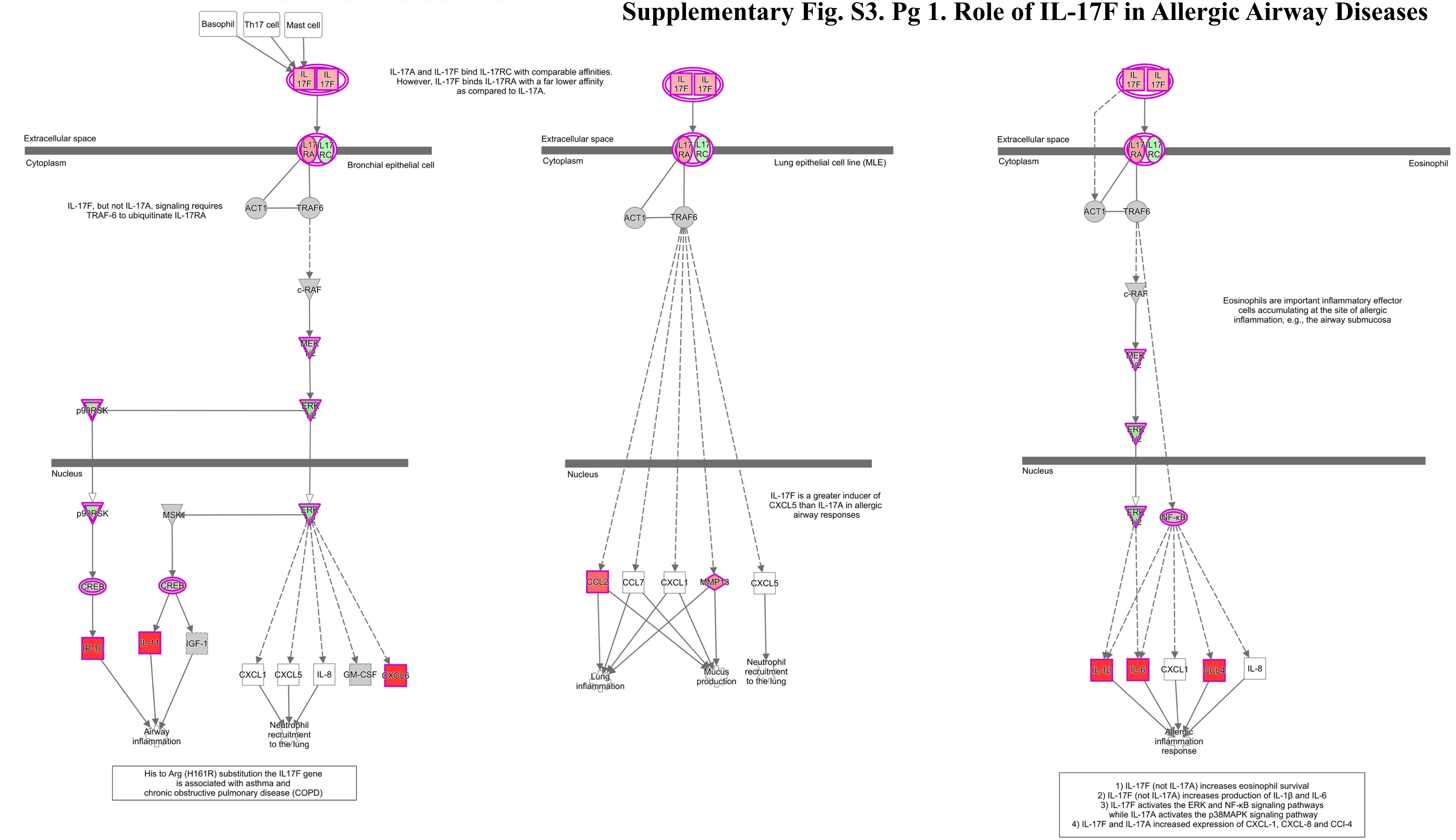

© 2000-2018 QIAGEN. All rights reserved.

Supplementary Fig. S3. Other upregulated pro-inflammatory pathways during ifosfamide induced hemorrhagic cystitis. Other major upregulated proinflammatory pathways associated with ifosfamide induced hemorrhagic cystitis were **Role of IL-17F in Allergic Airway Diseases**, p38 MAPK signaling, Leucocyte Extravasation signaling, HMGB1 signaling, TREM1 signaling. Pathways, Functional and Network analyses were generated using IPA[57]. For the key to the annotations, see description in Supplementary Fig. S2 legend.

p38 MAPK Signaling : Galaxy346-[Ifos_vs_Sal_DESeq2_result_file_on_data_21,_data_22,_and_others] : Expr Log Ratio

Supplementary Fig. S3. Pg 2. p38 MAPK signaling

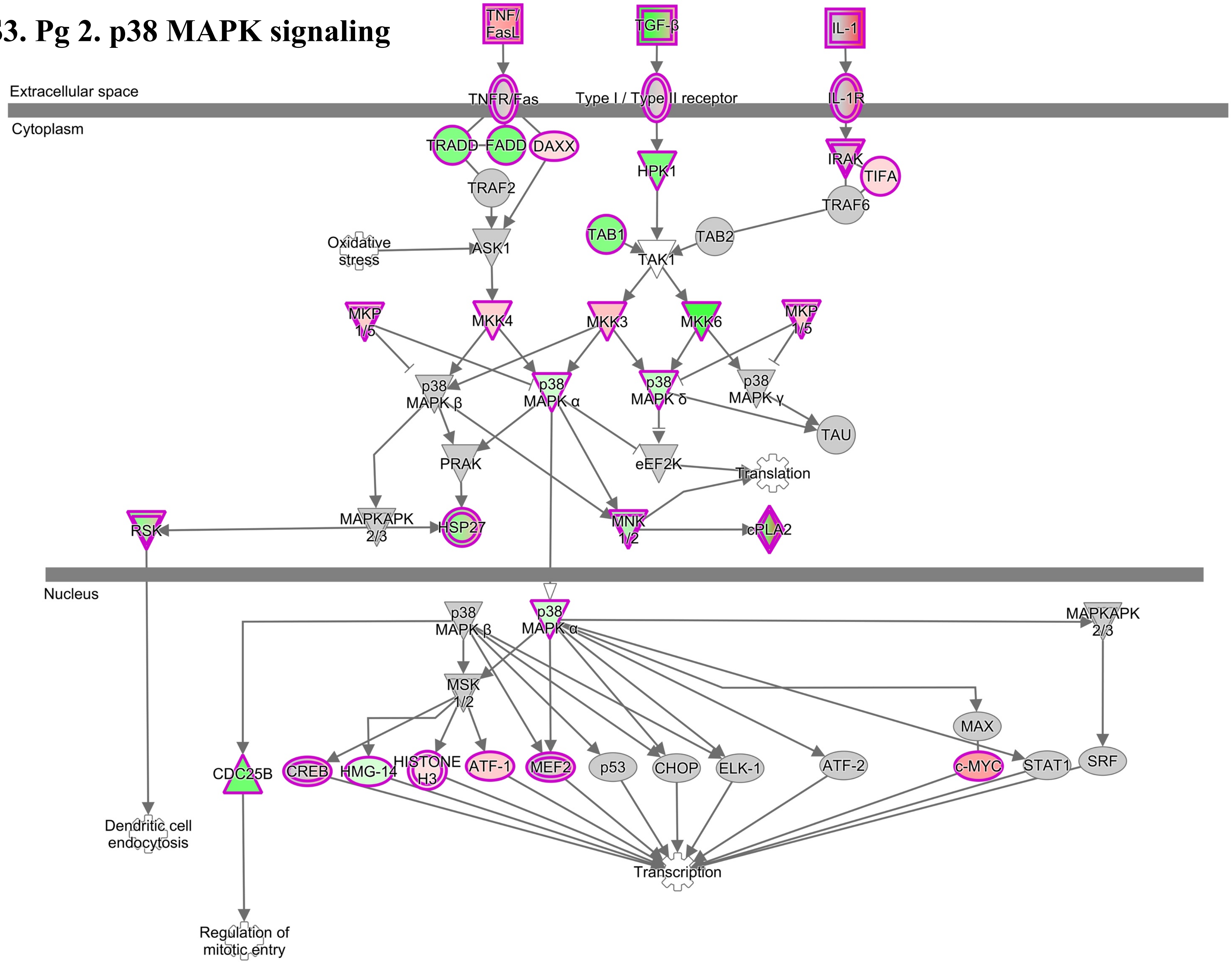

© 2000-2018 QIAGEN. All rights reserved.

Supplementary Fig. S3. Other upregulated pro-inflammatory pathways during ifosfamide induced hemorrhagic cystitis. Other major upregulated proinflammatory pathways associated with ifosfamide induced hemorrhagic cystitis were Role of IL-17F in Allergic Airway Diseases, **p38 MAPK signaling**, Leucocyte Extravasation signaling, HMGB1 signaling, TREM1 signaling. Pathways, Functional and Network analyses were generated using IPA[57]. For the key to the annotations, see description in Supplementary Fig. S2 legend.

Leukocyte Extravasation Signaling : Galaxy346-[Ifos_vs_Sal_DESeq2_result_file_on_data_21,_data_22,_and_others] : Expr Log Ratio

Supplementary Fig. S3. Pg 3. Leucocyte Extravasation signaling

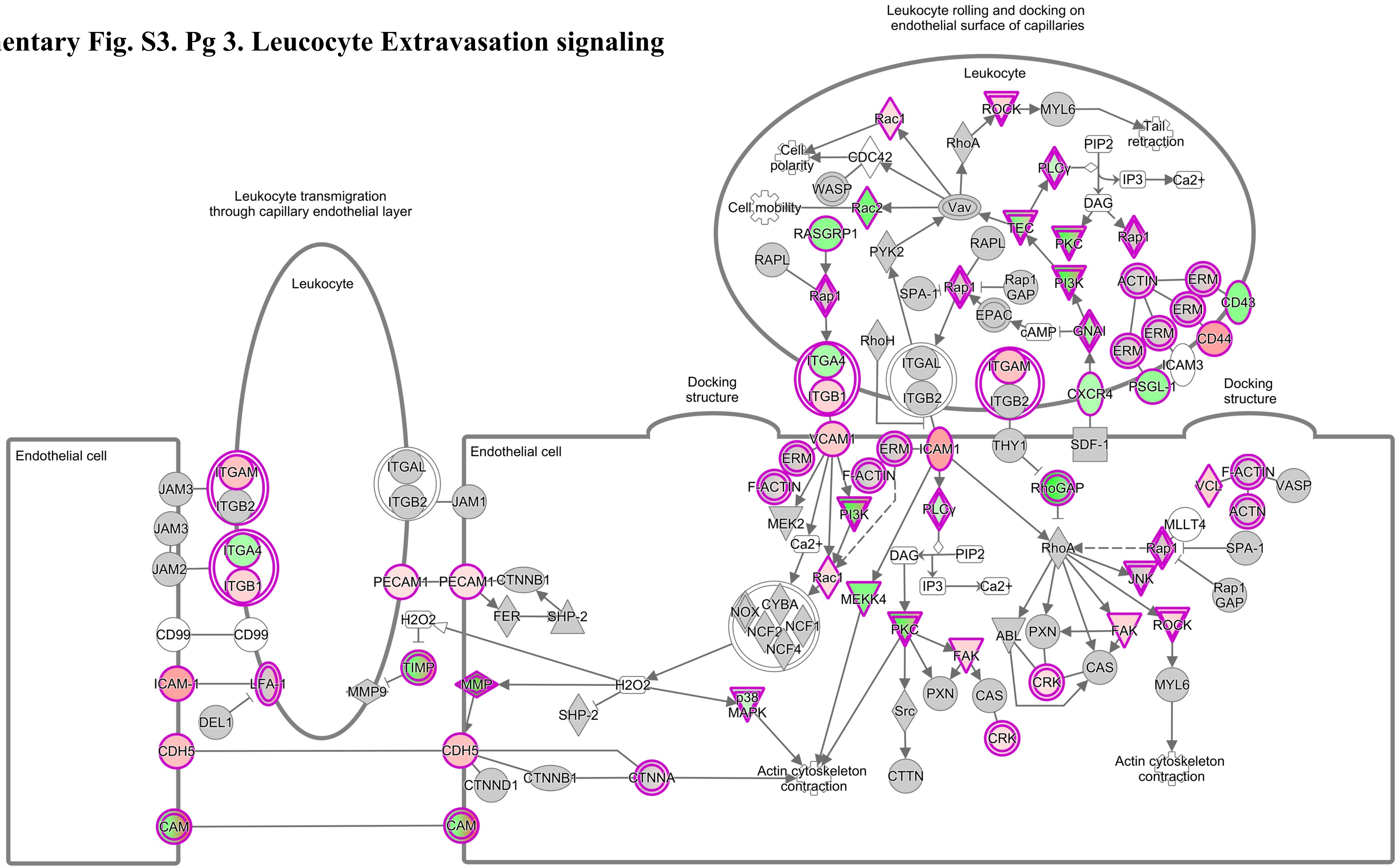

© 2000-2018 QIAGEN. All rights reserved.

Supplementary Fig. S3. Other upregulated pro-inflammatory pathways during ifosfamide induced hemorrhagic cystitis. Other major upregulated proinflammatory pathways associated with ifosfamide induced hemorrhagic cystitis were Role of IL-17F in Allergic Airway Diseases, p38 MAPK signaling, **Leucocyte Extravasation signaling**, HMGB1 signaling, TREM1 signaling. Pathways, Functional and Network analyses were generated using IPA[57]. For the key to the annotations, see description in Supplementary Fig. S2 legend.

HMGB1 Signaling : Galaxy346-[Ifos_vs_Sal_DESeq2_result_file_on_data_21,_data_22,_and_others] : Expr Log Ratio

Supplementary Fig. S3. Pg 4. HMGB1 signaling

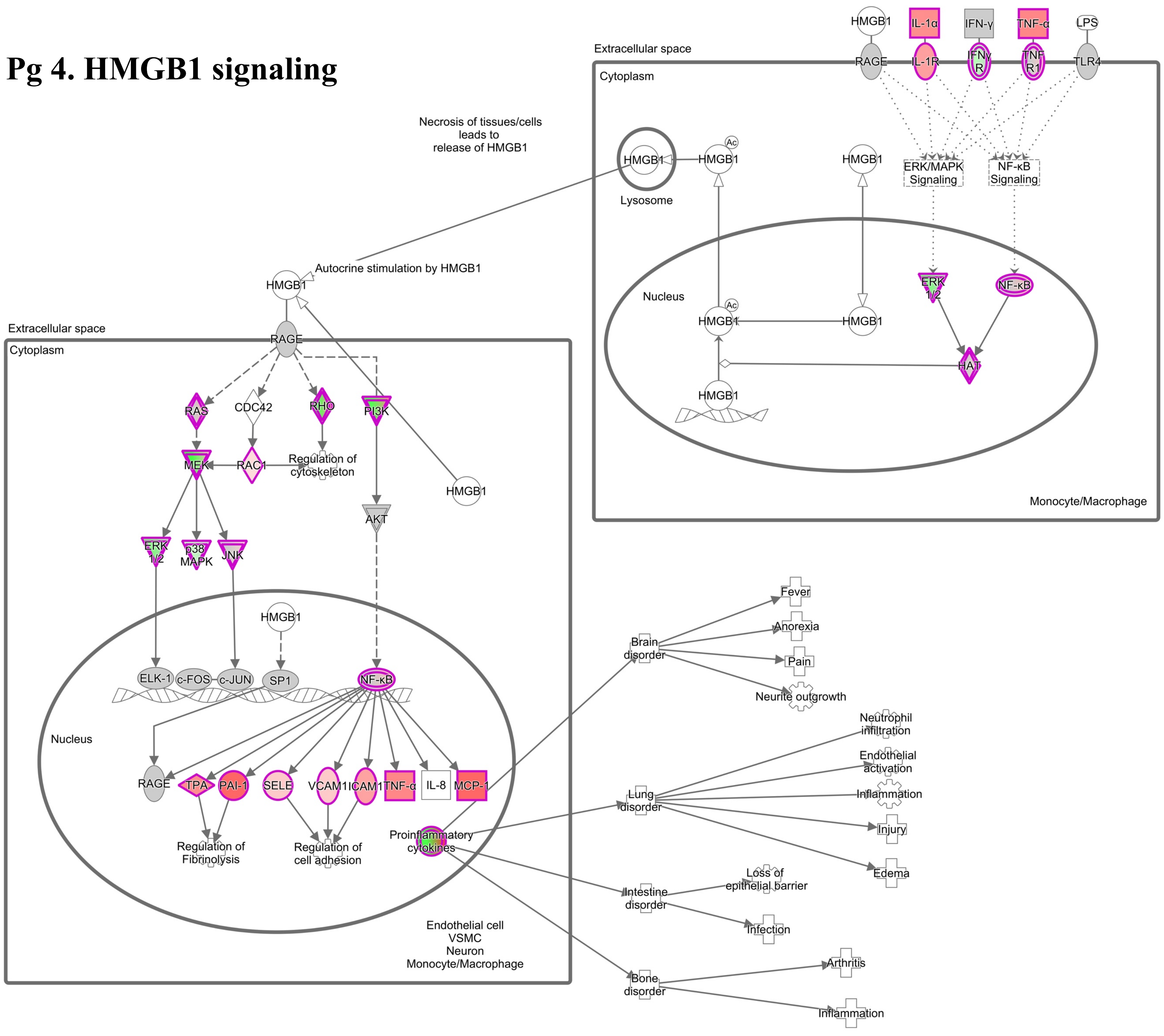

© 2000-2018 QIAGEN. All rights reserved.

Supplementary Fig. S3. Other upregulated pro-inflammatory pathways during ifosfamide induced hemorrhagic cystitis. Other major upregulated proinflammatory pathways associated with ifosfamide induced hemorrhagic cystitis were Role of IL-17F in Allergic Airway Diseases, p38 MAPK signaling, Leucocyte Extravasation signaling, **HMGB1 signaling,** TREM1 signaling. Pathways, Functional and Network analyses were generated using IPA[57]. For the key to the annotations, see description in Supplementary Fig. S2 legend.

Supplementary Fig. S3. Pg 5: TREM1 signaling.

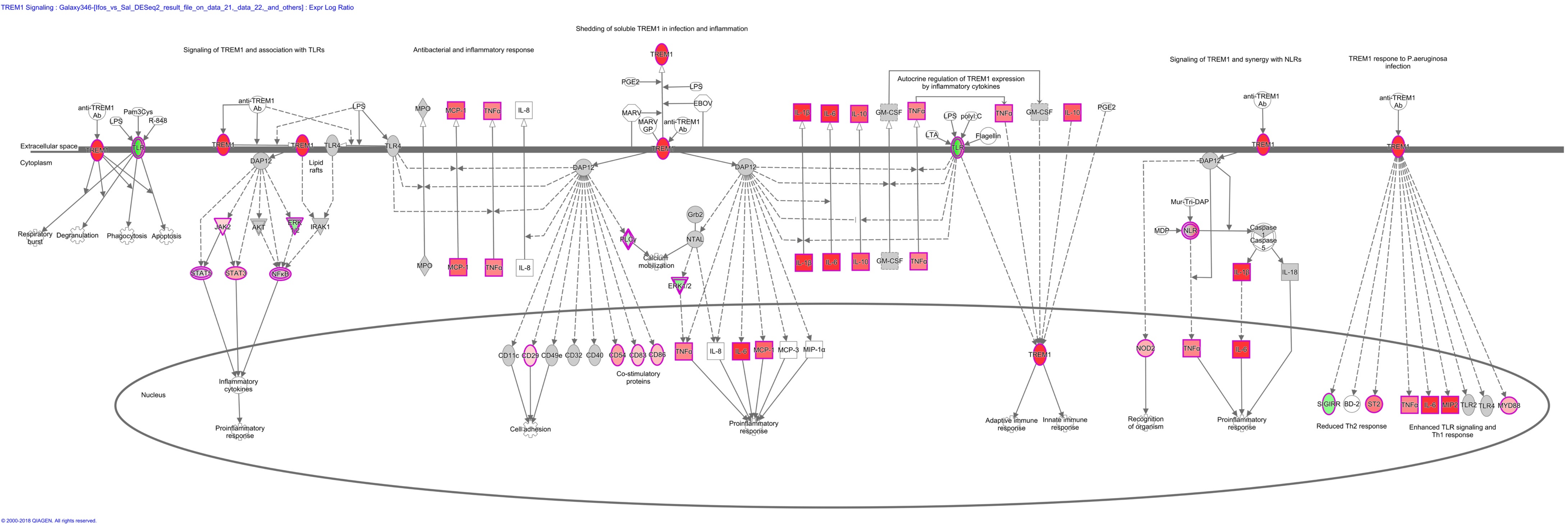

Supplementary Fig. S3. Other upregulated pro-inflammatory pathways during ifosfamide induced hemorrhagic cystitis. Other major upregulated proinflammatory pathways associated with ifosfamide induced hemorrhagic cystitis were Role of IL-17F in Allergic Airway Diseases, p38 MAPK signaling, Leucocyte Extravasation signaling, HMGB1 signaling, **TREM1 signaling.** Pathways, Functional and Network analyses were generated using IPA[57]. For the key to the annotations, see description in Supplementary Fig. S2 legend.

Supplementary Fig. S4. Pg 1

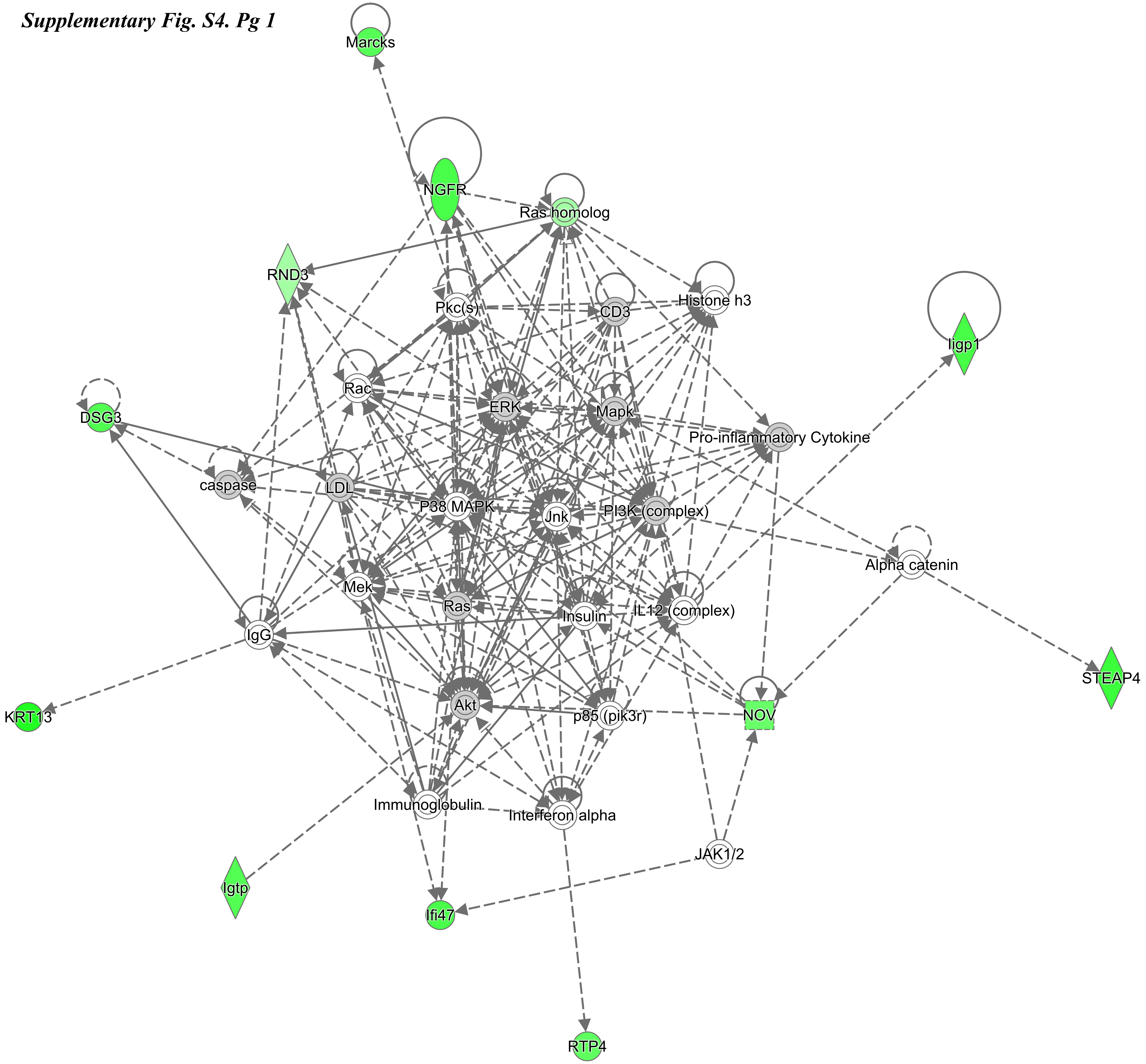

Supplementary Fig. S4. Mechanistic network analysis. Pathways, Functional and Network analyses were generated using IPA[57]. Mechanistic network analysis of transcriptome of IPSE pretreated mice compared to ifosfamide only mice showed downregulation of interactions between several proinflammatory genes. In addition to the network interaction between chemokines and interferon induced proteins, we also recorded more downregulatory mechanistic network interaction between genes encoding interferons induced proteins. Orange color signifies directional gene expression that is part of dataset, passed the cut off values and upregulated. Green color signifies directional gene expression that is part of dataset, passed the cut off values and downregulated. Gray color signifies gene expression that is part of dataset but unchanged. White color signifies genes that is not part of dataset. For other keys to the shape annotations, see description in Supplementary Fig. S2 legend.

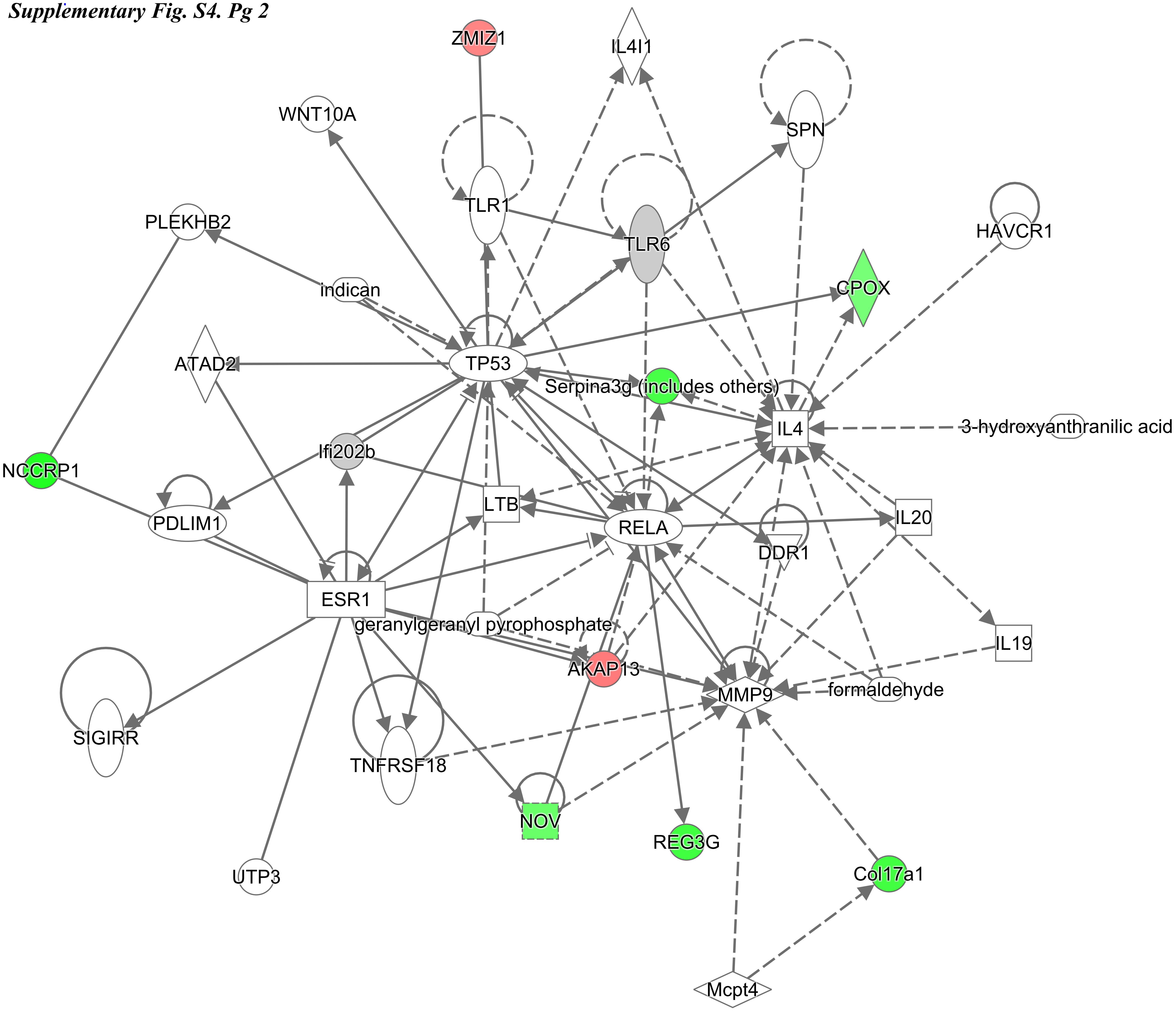

Supplementary Fig. S4. Mechanistic network analysis. Pathways, Functional and Network analyses were generated using IPA[57]. Mechanistic network analysis of transcriptome of IPSE pretreated mice compared to ifosfamide only mice showed downregulation of interactions between several proinflammatory genes. In addition to the network interaction between chemokines and interferon induced proteins, we also recorded more downregulatory mechanistic network interaction between genes encoding interferons induced proteins. Orange color signifies directional gene expression that is part of dataset, passed the cut off values and upregulated. Green color signifies directional gene expression that is part of dataset, passed the cut off values and downregulated. Gray color signifies gene expression that is part of dataset but unchanged. White color signifies genes that is not part of dataset. For other keys to the shape annotations, see description in Supplementary Fig. S2 legend.

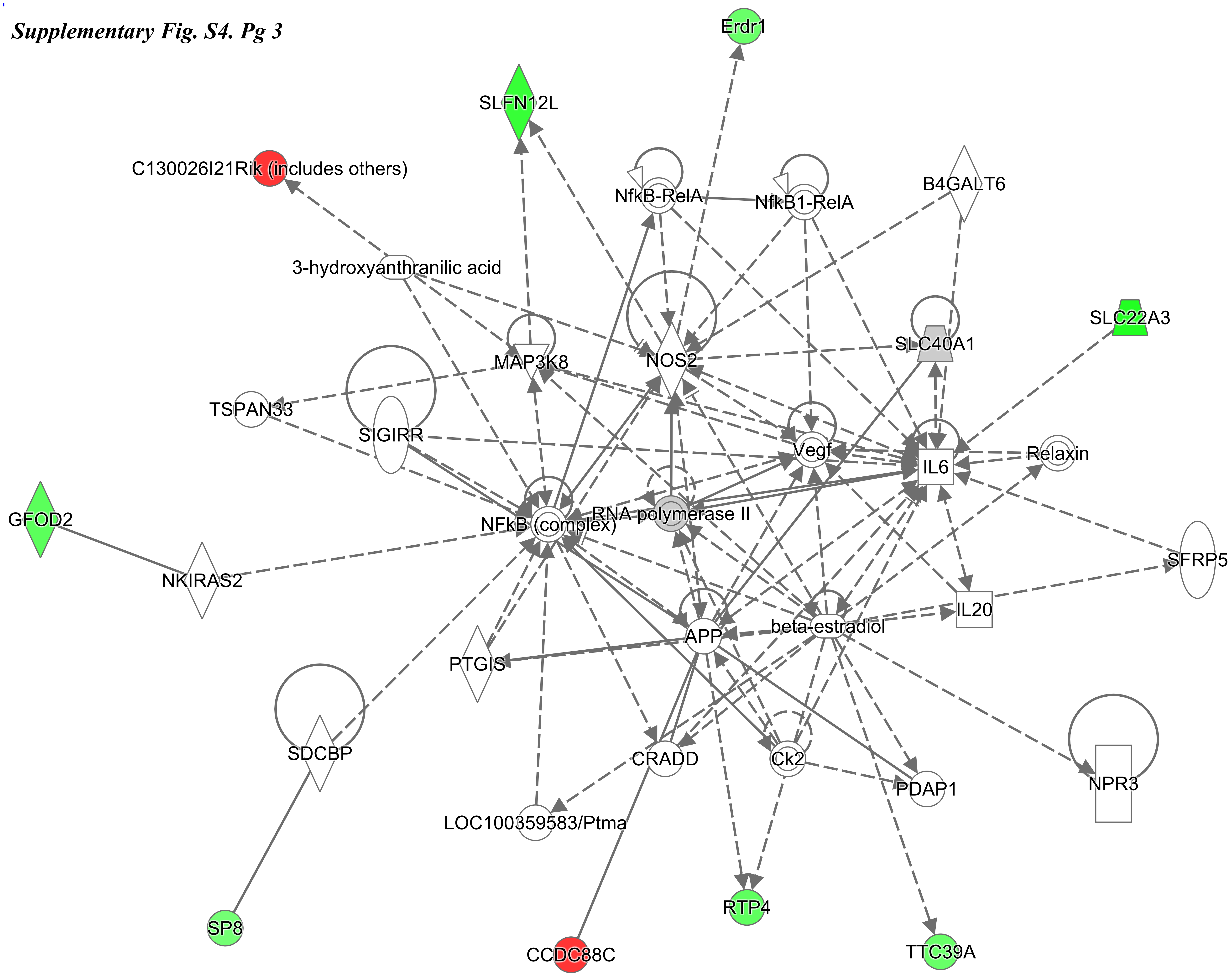

Supplementary Fig. S4. Mechanistic network analysis. Pathways, Functional and Network analyses were generated using IPA[57]. Mechanistic network analysis of transcriptome of IPSE pretreated mice compared to ifosfamide only mice showed downregulation of interactions between several proinflammatory genes. In addition to the network interaction between chemokines and interferon induced proteins, we also recorded more downregulatory mechanistic network interaction between genes encoding interferons induced proteins. Orange color signifies directional gene expression that is part of dataset, passed the cut off values and upregulated. Green color signifies directional gene expression that is part of dataset, passed the cut off values and downregulated. Gray color signifies gene expression that is part of dataset but unchanged. White color signifies genes that is not part of dataset. For other keys to the shape annotations, see description in Supplementary Fig. S2 legend.

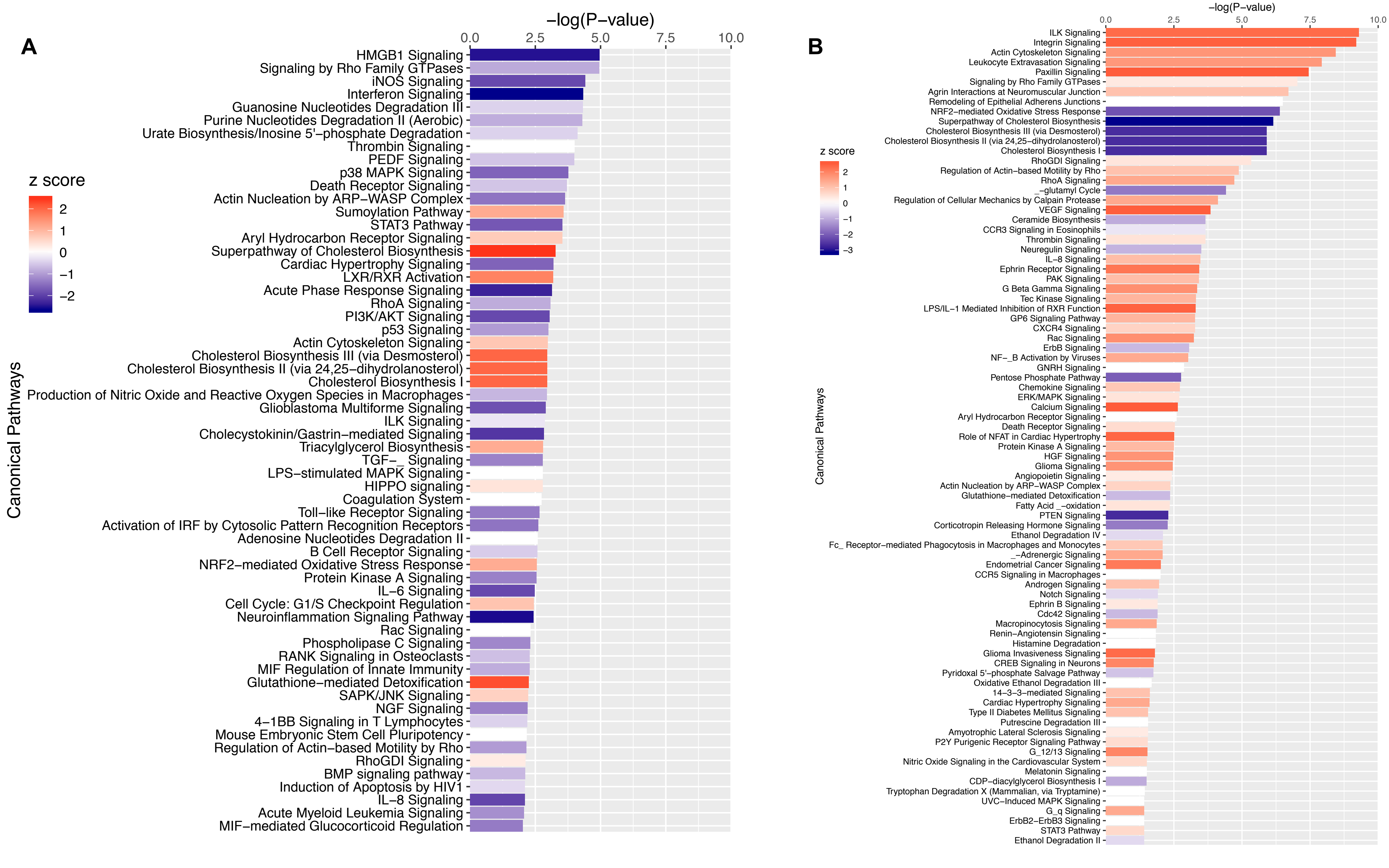

Supplementary Fig. S5. Differentially altered pathways in bladders of mice challenged with ifosfamide with pretreatment with IPSE variants. Mice were pretreated with saline, IPSE or IPSE NLS mutant, 24 hours before challenge with 400mg/kg of ifosfamide. The bladders were subjected to transcriptional profiling (RNA-Seq) and functional analysis using IPA[57]. . (A) Differentially altered genes by pretreatment with IPSE NLS mutant compared to the ifosfamide only group. (B) Differentially altered genes by pretreatment with IPSE NLS mutant compared to pretreatment with wild type IPSE, both before ifosfamide challenge. Bars are colored according to z-score (predicts activation or inhibition based on the degree of overlap between directional expressions from observed data and the QIAGEN-curated public knowledge base), with red showing activation and blue denoting inhibition. The size of each bar is proportional to its –log(p-value).

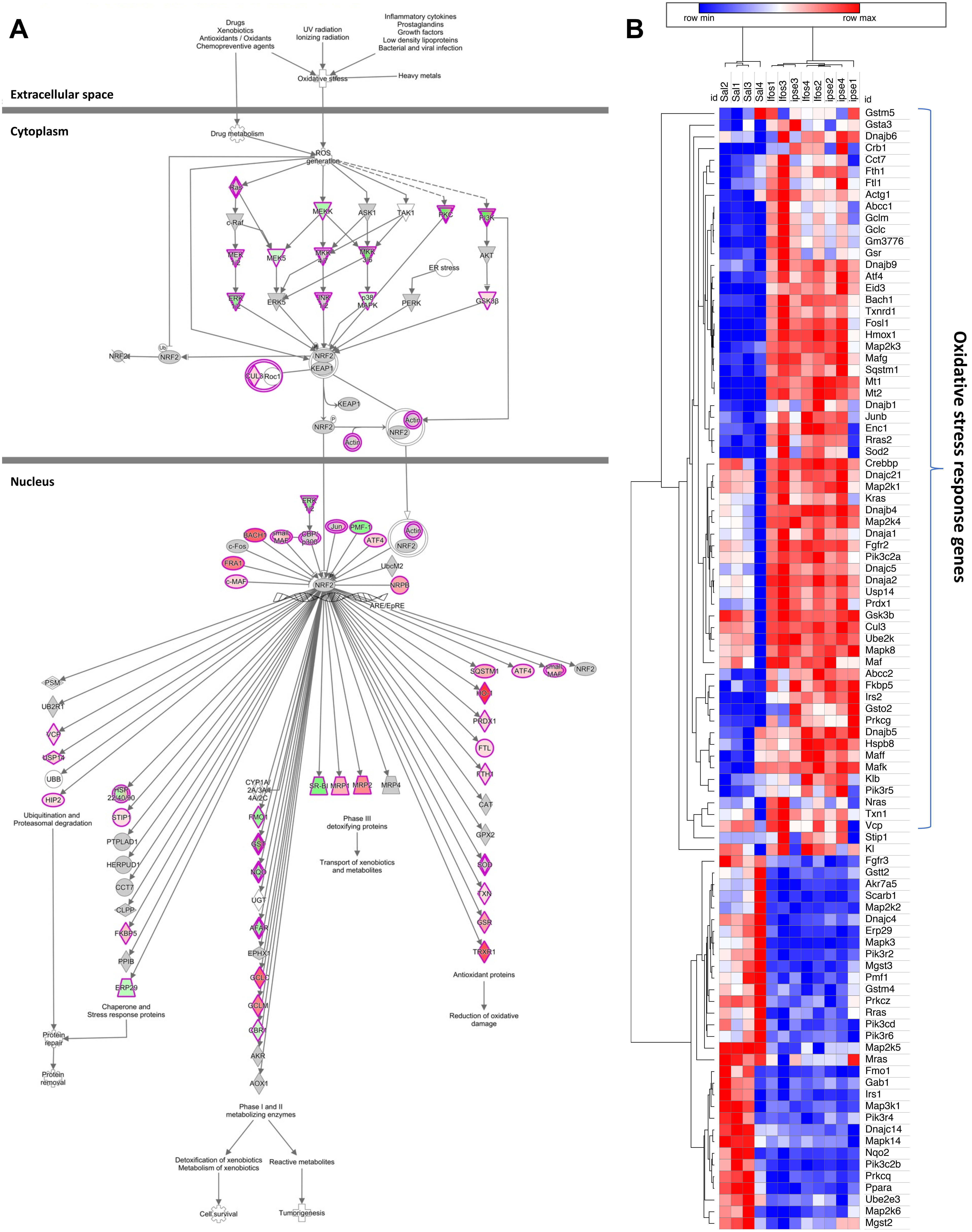

Supplementary Fig. S6. NRF2 mediated oxidative stress responses pathway. NRF2 is the major pathway regulating response to oxidative stress. It induces the expression of heme oxygenase pathway, the first enzyme of the heme homoeostasis pathway, and the expression of several antioxidant enzymes and proteins. An abridged version of this figure is shown in Fig. 7. Pathways, Functional and Network analyses were generated using IPA[57]. For the key to the annotations, see description in Supplementary Fig. S2 legend.

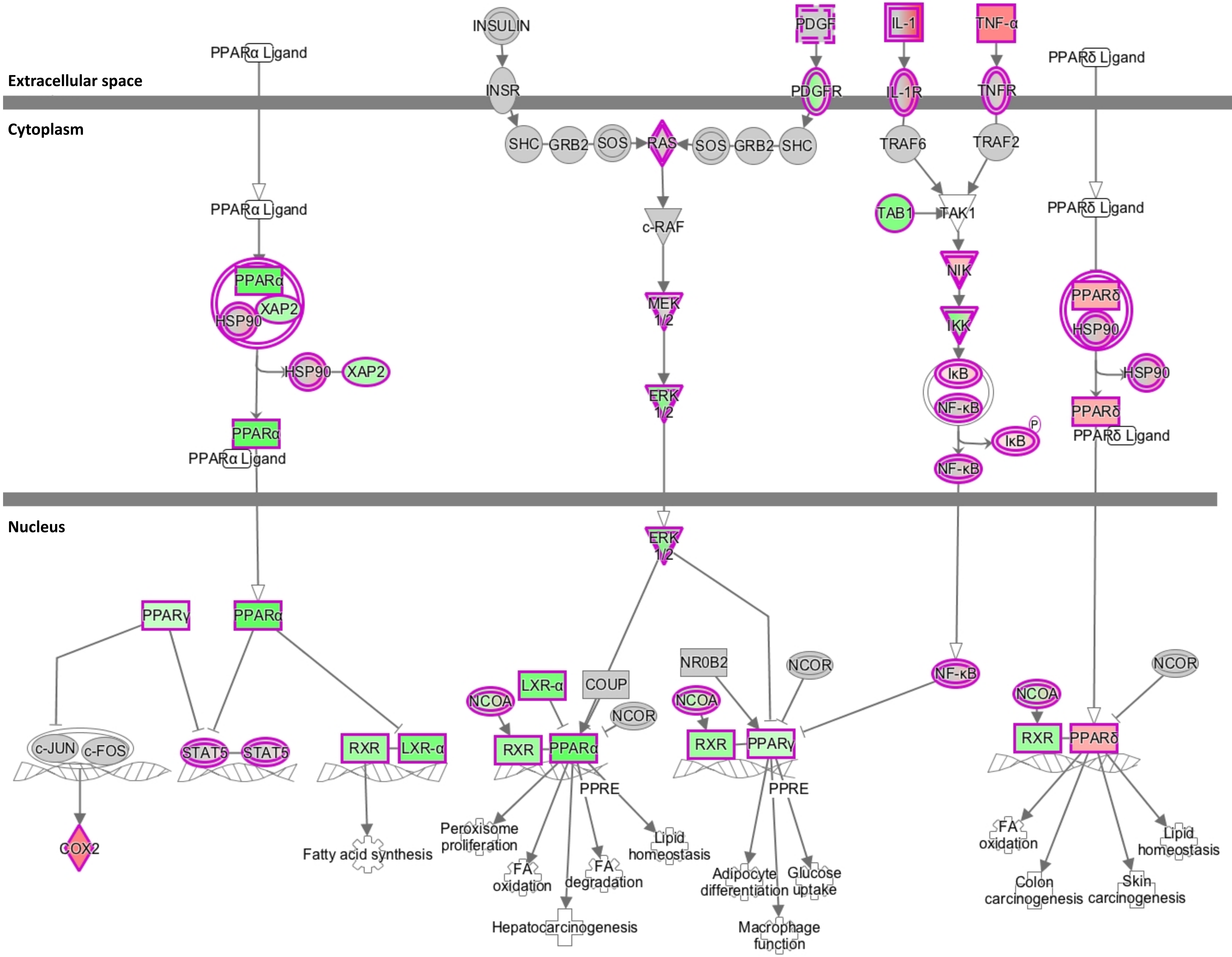

Supplementary Fig. S7. PPAR signaling pathway. PPAR cascade is the major pathway regulating lipid homoeostasis. PPAR has been shown to play an anti-inflammatory role [44], thus, here downregulated in response to ifosfamide induced cystitis. Pathways, Functional and Network analyses were generated using IPA[57]. For the key to the annotations, see description in Supplementary Fig. S2 legend.

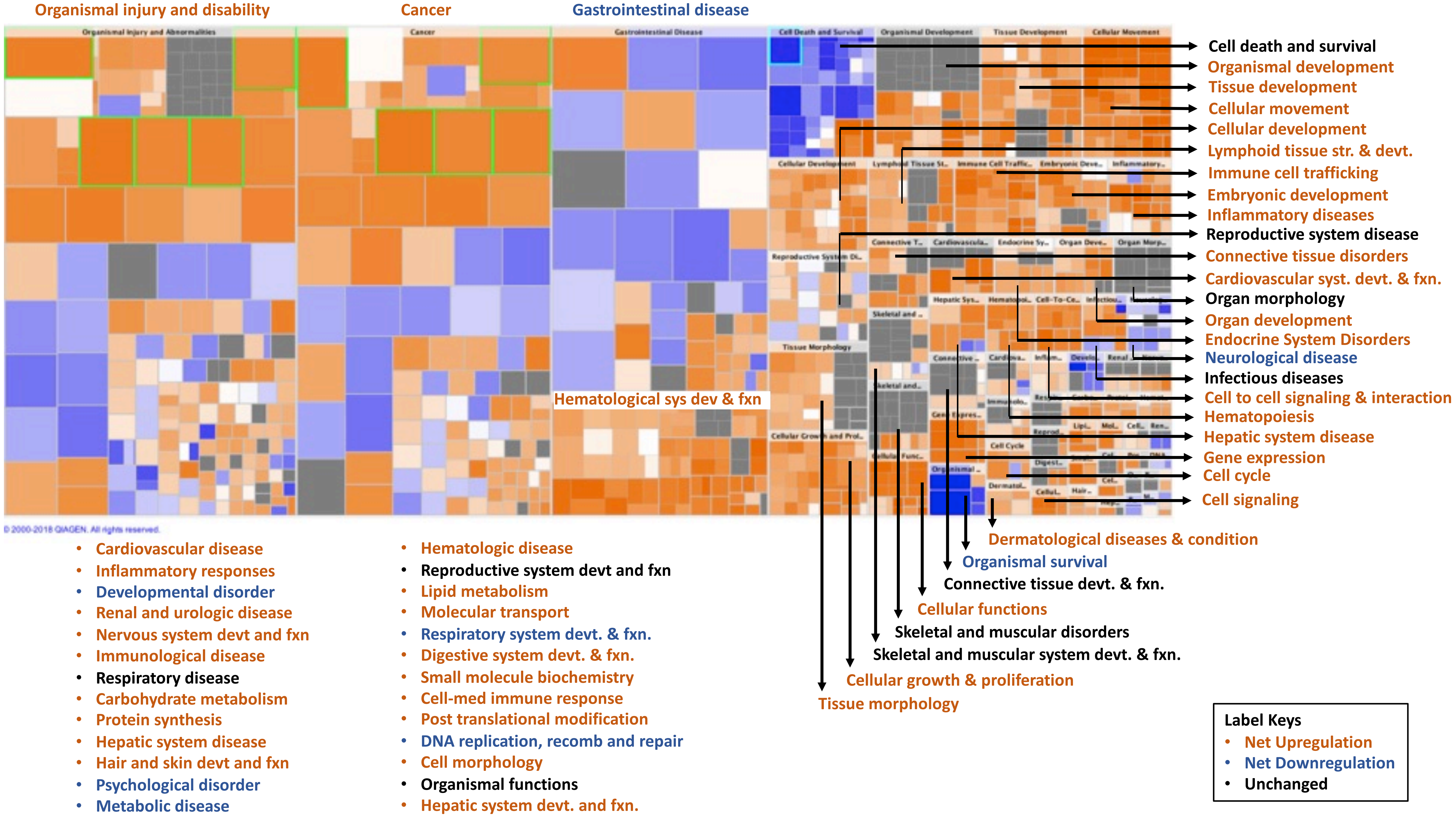

Supplementary Fig. S8. Diseases and Function Tree map. This is a graphical representation of changes in the diseases and disorders, molecular and cellular functions, physiological system development and functions altered due to ifosfamide induced cystitis. We saw high upregulation of functions related to organismal injury and abnormalities, inflammatory diseases, cancer, cell proliferation, cellular movement and hematological systems development and function, and downregulation of cell death in response to ifosfamide induced cystitis. Pathways, Functional and Network analyses were generated using IPA[57].

Neuroinflammation involves numerous cell types, acts to clear neuronal damage, and plays a key role in maintaining the homeostasis of CNS.
Homeostasis can be lost through various regulatory failures, or when humoral immune components cross the blood-brain barrier, causing chronic inflammation with excessive cell and tissue damage, which is associated with neurodegenerative diseases.

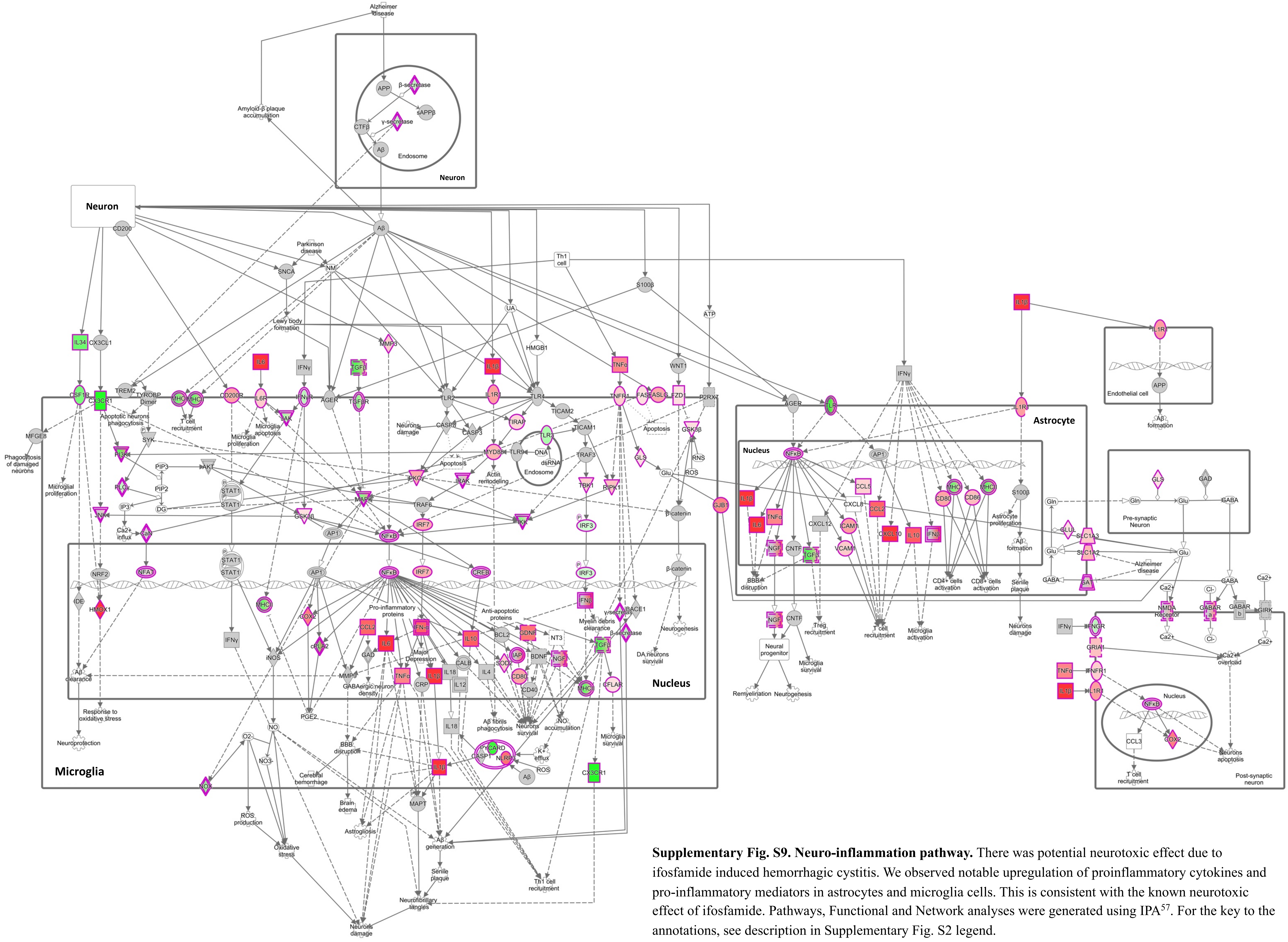

Supplementary Fig. S9. Neuro-inflammation pathway. There was potential neurotoxic effect due to ifosfamide induced hemorrhagic cystitis. We observed notable upregulation of proinflammatory cytokines and pro-inflammatory mediators in astrocytes and microglia cells. This is consistent with the known neurotoxic effect of ifosfamide. Pathways, Functional and Network analyses were generated using IPA[57]. For the key to the annotations, see description in Supplementary Fig. S2 legend.

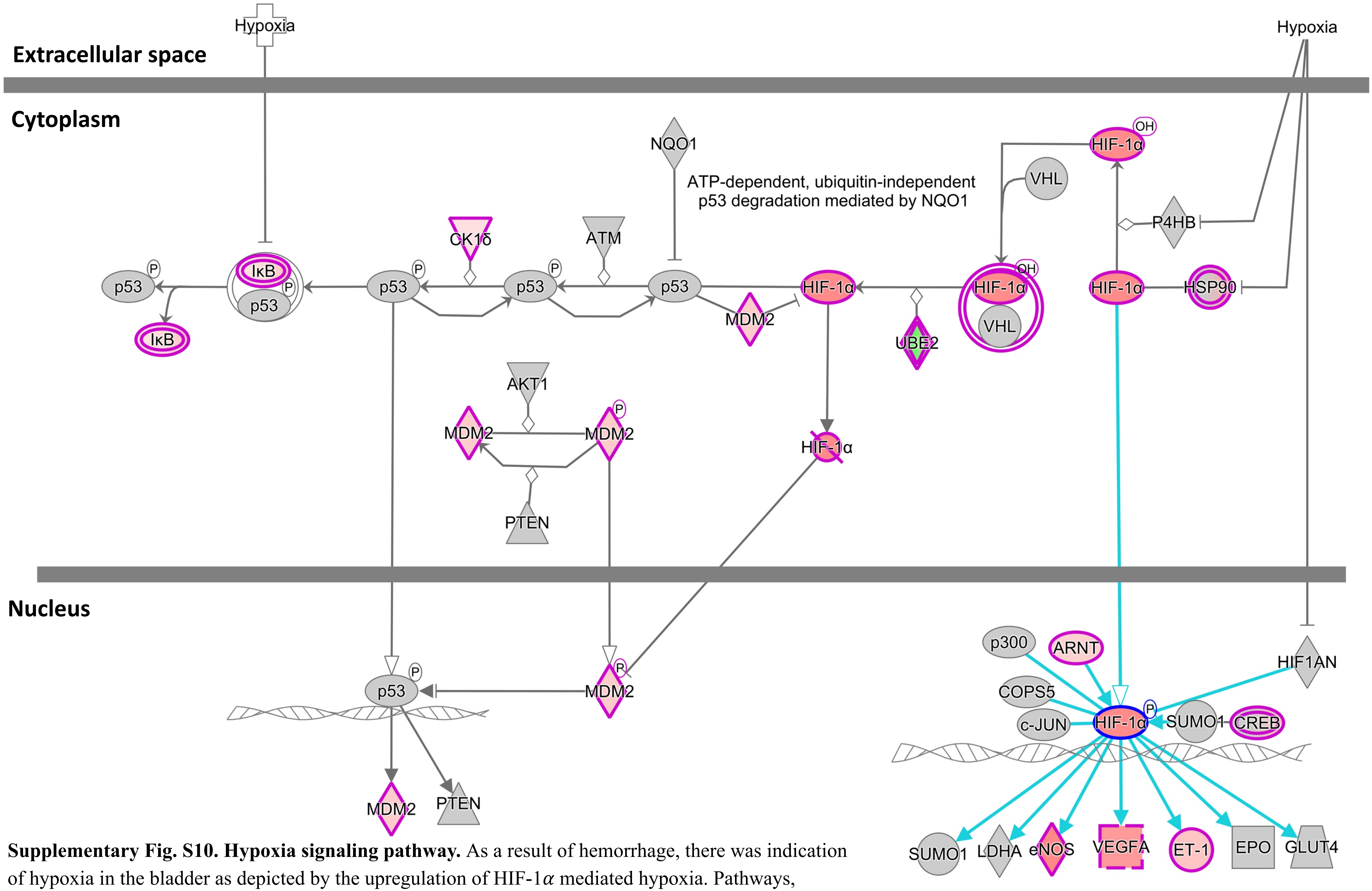

Supplementary Fig. S10. Hypoxia signaling pathway. As a result of hemorrhage, there was indication of hypoxia in the bladder as depicted by the upregulation of HIF-1α mediated hypoxia. Pathways, Functional and Network analyses were generated using IPA[57]. For the key to the annotations, see description in Supplementary Fig. S2 legend.
